# Bioprospecting the thermal waters of the Roman baths: isolation of oleaginous species and analysis of the FAME profile for biodiesel production

**DOI:** 10.1186/2191-0855-3-9

**Published:** 2013-01-31

**Authors:** Holly D Smith-Bädorf, Christopher J Chuck, Kirsty R Mokebo, Heather MacDonald, Matthew G Davidson, Rod J Scott

**Affiliations:** 1 Department of Biology and Biochemistry, University of Bath, Bath, BA2 7AY, UK; 2 Department of Chemical Engineering, University of Bath, Bath, BA2 7AY, UK; 3 Department of Chemistry, University of Bath, Bath, BA2 7AY, UK; 4 Department of Applied Sciences, University of the West of England, Bristol, BS16 1QY, UK

**Keywords:** Biodiesel, Microalgae, Cyanobacteria, Biofuel

## Abstract

The extensive diversity of microalgae provides an opportunity to undertake bioprospecting for species possessing features suited to commercial scale cultivation. The outdoor cultivation of microalgae is subject to extreme temperature fluctuations; temperature tolerant microalgae would help mitigate this problem. The waters of the Roman Baths, which have a temperature range between 39°C and 46°C, were sampled for microalgae. A total of 3 green algae, 1 diatom and 4 cyanobacterial species were successfully isolated into ‘unialgal’ culture. Four isolates were filamentous, which could prove advantageous for low energy dewatering of cultures using filtration.

Lipid content, profiles and growth rates of the isolates were examined at temperatures of 20, 30, 40°C, with and without nitrogen starvation and compared against the oil producing green algal species, *Chlorella emersonii*. Some isolates synthesized high levels of lipids, however, all were most productive at temperatures lower than those of the Roman Baths. The eukaryotic algae accumulated a range of saturated and polyunsaturated FAMEs and all isolates generally showed higher lipid accumulation under nitrogen deficient conditions (*Klebsormidium* sp. increasing from 1.9% to 16.0% and *Hantzschia* sp. from 31.9 to 40.5%). The cyanobacteria typically accumulated a narrower range of FAMEs that were mostly saturated, but were capable of accumulating a larger quantity of lipid as a proportion of dry weight (*M. laminosus,* 37.8% fully saturated FAMEs). The maximum productivity of all the isolates was not determined in the current work and will require further effort to optimise key variables such as light intensity and media composition.

## Introduction

'The transport fuel sector constitutes a large proportion of global energy demand but renewable alternatives, in comparison to other energy sectors, are largely underdeveloped (Schenk et al. 2008). To be competitive renewable liquid fuels must match the performance, energy density and versatility of current fossil fuels (Rupprecht 2009). It is therefore imperative that renewable, energy dense liquid fuels capable of integration into the existing infrastructure are rapidly developed.

One renewable liquid fuel is biodiesel, comprised of the fatty acid methyl esters (FAMEs) produced from the esterification of biologically derived oils. Biodiesel contains chain lengths between C_14_-C_24_ with varying degrees of unsaturation (Varfolomeev and Wasserman, 2011). The FAME profile is also dependent on the specific producing organism as well as its growing conditions (Saraf and Thomas, 2007). Thus, biodiesel tends to have variable fuel properties that can substantially affect engine performance (Fortman et al. 2008). Biodiesel contains a relatively high oxygen content by weight which results in more complete combustion than mineral diesel resulting in lower CO, particulate matter and hydrocarbon emissions (Song et al., 2008). Currently biodiesel is produced from agricultural crops such as rapeseed, soybean or palm that occupy limited agricultural resources, are produced on a seasonal basis and in most cases generate a low energy return (Rittmann, 2008). They also pose a threat to biodiversity, as marginal land is often ecologically valuable, constituting habitat or acting as a watershed (Gressel, 2008).

In an attempt to replace these first generation feedstocks, there has been a resurgence of interest in developing oleaginous microalgae. Algal biofuels have potentially high energy returns and a small ecological footprint (Groom et al. 2008), though the FAME profile is reportedly more variable than those produced by terrestrial crops (Haik et al. 2011). Microalgae have the potential for all year round harvest (Chisti, 2008), are lipid-rich and offer the prospect of harnessing the residual biomass in conventional biomass technologies (Um and Kim, 2009). Algae culture can be coupled to industrial CO_2_ sequestration (Chisti, 2007), bioremediation or scrubbing of waste streams (Pittman et al. 2011). In addition, algae are capable of producing a multitude of other high-value products including biopolymers, vitamins, antioxidants, polysaccharides, proteins, and pharmaceuticals (Raja et al., 2008). Economic analysis demonstrates that co-production of these products is currently essential for commercial viability (Ono and Cuello, 2006).

Algae are responsible for 50% of global CO_2_ fixation and have colonised diverse ecological habitats from oceans to hot springs, snowfields and waste streams (Croft et al. 2006). Microalgae are best described as unicellular, microscopic (2-200μm) photosynthetic organisms as they have both prokaryotic (cyanobacteria) and eukaryotic representatives, with over 300,000 species (Pulz and Gross, 2004). The most abundant microalgae in any given environment are generally the diatoms, green algae, cyanobacteria and golden algae (Mutanda et al. 2011).

Microalgae which can accumulate lipids at over 20% of their dry weight are referred to as oleaginous species, most of which belong to the green algae and diatoms (Pulz and Gross, 2004). Cyanobacteria are also capable of accumulating lipid, yet are comparatively understudied with regard to lipid content and profile (Karatay and Dönmez 2011). *Chlorella* spp. is a commonly studied oleaginous genera with a very high CO_2_ fixation rate and lipid content compared to other green algae. In the long term, genetic modification might well have the biggest impact on realising fuels from microalgae but algal bioengineering is in its infancy (Chisti and Yan, 2011; Purton and Stevens, 1997). In addition, transgenic cells usually exhibit lower fitness as well as there being restrictions and public concern over their commercial use, therefore, genetic modification must ‘compliment and not substitute screening of new species’ (Pulz and Gross, 2004).

Since microalgae are a diverse group of organisms, with large variations in growth requirements, growth rate, fatty acid content and the ability to produce other valuable products, the screening and isolation of new algal species is an important goal in developing algal biofuels (Hu et al. 2008). Choice of species is an essential consideration for any applied microalgal biotechnology (Ratha and Prasanna, 2012). The dilute nature of microalgal cultures means extensive dewatering is required prior to biomass processing, which significantly raises energy costs using current methods such as centrifugation, electrophoresis and floatation (Chisti and Yan, 2011). Harvesting costs are therefore an important consideration when screening for new strains, as differences in morphology can have a substantial impact on the cost of production (Mutanda et al. 2011). Whilst low energy methods such as filtration are challenging with unicellular microalgal species these can be very effective for filamentous species (Uduman et al. 2010 and Mohan et al. 2010).

Bioprospecting of diverse aquatic environments offers a way to obtain new oleaginous microalgae with features suited to commercialisation. Hypersaline and thermophillic springs have proved ideal environments for the identification novel microalgae (Mutanda et al. 2011). Extremophilic algae may be beneficial for industrial applications, as the unique environment minimizes contamination risk, particularly in open pond systems, and can provide buffering against fluctuations in temperature (Pulz and Gross, 2004). Significantly, the most productive commercially cultivated species could be considered extremophillic (Xu et al. 2009).

Commonly cultured microalgal species have optimal growth between 16-27°C, with temperatures below 16°C resulting in slow growth, and temperatures above 35°C often being lethal (Barsanti and Gualtieri, 2006). Although thermotolerant microalgal species have been isolated from hot springs (primarily in the USA and Japan), few have been extensively studied to date (Ratha and Prasanna, 2012).

The largest geothermal spring in the UK is situated in the city of Bath at the Roman Baths (ST750647) (Atkinson and Davison, 2002). A series of 3 thermal springs first supply water at a rate 720 l h^-1^ to the Kings Bath (46.5°C), which then drains into the Great Bath 39.0°C; (Andrews et al. 1982). The baths were gradually constructed by Roman settlers between 70-370 AD, but fell into disrepair gradually becoming buried during the 5th century as the Romans withdrew from Britain (pers. comm. Tom Byrne). Excavation of the Baths began in 1878 and have remained open to the elements for the last 130 years (Kellaway, 1991). Since thermotolerance is attractive for biofuel production we set out to bioprospect the hot waters of the Roman Baths for microalgal strains with an ability to produce lipids for biodiesel production.

## Materials and methods

### Sampling of bath water for algae

Samples of water (1l) were taken from the Great Bath and the Kings Bath and analysed by Severn Trent Services, the pH and temperature were measured on site. Three visible filamentous algae were sampled from scrapings taken from microbial mats submerged beneath the water surface. Approximately 90 l of water from the Great Bath was filtered through a custom-made filter-stack at 3 positions around the Great Bath (the water entry point, exit point and opposite). The custom filter stack comprised of an acrylic tube with a coarse 3 mm mesh metal filter, a sponge filter and a Whatman GF fine filter paper. The GF filter paper layer was used as a sample for further culturing.

### Establishing unialgal cultures

The algae loaded GF filter papers were immediately cultured using a method adapted from Ferris and Hirsch (1991) (‘filter paper method’). Filters were placed on top of a stack of 5 Whatman GF papers saturated with a mixture of 1l BBM:BG media 1:1, inside a glass petridish with lid, before sealing with Parafilm. Samples were then left at room temperature and in low light (on lab bench) until colonies appeared.

Small samples were taken from colonies and placed on a microscope slide with a coverslip for quick examination under microscope Nikon Eclipse 90i and confocal D-Eclipse. Colonies with green or blue-green pigmentation (either single celled, filamentous or of diatom morphology) were picked off, diluted and re-plated on filter paper as described above, as well as on BBM pH 6.5 and BG-11 pH 7.1 agar plates (1% agar). Where possible, both filter paper and agar methods were repeated until a unialgal culture was derived from single colonies comprised of one alga (assessed under inverted microscope Nikon eclipse TE2000-S).

The method of (Vaara et al. 1979) was used (‘agar plate scoring method’) to clean filamentous samples of contaminant fungal species. Agar plates were scored perpendicular to the end of inoculation. Plates were covered in black cloth with the end opposite the inoculation site exposed to a light source. This arrangement encourages growth of algal filaments along the agar scores, towards the light, thereby shedding fungal and bacterial contaminants.

All isolates were deposited in the CCAP culture collection and have been assigned the following collection numbers*. Oscillatoria sancta* (CCAP 1459/46), *Microcoleus chthonoplastes* (CCAP 1449/2), *Mastigocladus laminosus* (CCAP 1447/3), *Klebsormidium sp.* (CCAP 335/20), *Coelastrella saipanensis* (CCAP 217/9), *Chroococcidiopsis thermalis* (CCAP 1423/1) and *Hantzschia sp.* (CCAP 1030/1).

### Examining temperature tolerance

An experiment to determine a suitable temperature range for further analysis was carried out using heat blocks. All isolates together with a reference alga, *Chlorella emersonii* (*Ce)*, were cultivated in triplicate in 15 ml Sterilin falcon tubes at temperatures between 25-60°C at intervals of 5°C. Tubes were shaken manually twice a day and the lids opened once a day to allow for gas exchange. The growth was assessed visually by comparing the tubes across the temperature ranges. As a result of the experiment 20°C, 30°C and 40°C were chosen for further experiments.

Each species was cultivated in 2 × 250ml conical flasks with 100 ml of suitable media: Bolds Basal Medium pH 6.5 (BBM) for eukaryotes and Blue Green-11 Medium pH 7.1 (BG-11) for prokaryotes and a ‘Diatom Media’ for the diatom (DM), comprised of BBM with the addition of 100μl B vitamins (0.0024% B_1_B_7_B_12_ 1:1:1) and 100μl sodium silicate. The flasks were inoculated with 100μl stock cultures. Cultures were incubated in a plant growth chamber (in Sanyo MLR-351) on a rotary platform (Sanyo MIR-S100) at 100 rpm, with a 100-150 μmol m^-2^ s^-1^ of light on a 12 hour light/dark cycle. *Chroococcidiopsis thermalis* and *Hantzschia* sp. culture flasks were covered in a double layer of neutral density filter (StageElectrics, Bristol, UK) due to their sensitivity to light.

After 12 days one flask of each culture was pelleted and re-suspended in an appropriate nitrogen free media and cultivated for a further 6 days. Samples were then pelleted by centrifugation and lyophilised.

For single celled algae, growth measurements were recorded as cell counts using a haemocytometer and dry weights calculated by lyophilising 2 ml of the culture. Filamentous algae had a tendency to form mats or aggregates in the flasks and to stick to the walls of the vessel. Consequently, growth measurements for filamentous algae were done by initiating cultures in pre-weighed 15 ml falcon tubes containing 7.5 ml media. The algae were continuously mixed using a bloodtube rotator (Stuart Scientific SB1), Stone, Staffordshire, UK. At 3 day intervals 3 tubes were removed, centrifuged to remove water and lyophilised.

### Identification of isolates by DNA barcoding

Algal cultures were grown in 100 ml of appropriate liquid media under culture conditions described above. DNA extracted from 50 ml of each culture was centrifuged and the wet pellet transferred to a 1.5 ml Eppendorf. 300 μl of 20 mM Na-phosphate buffer (pH 8), 150 μl of lysis solution (10% sodium dodecyl sulphate, 0.5M Tris HCl (pH 8), 0.1M NaCl) and sufficient 0.1 mm silica/zirconium beads added to make a paste which was then ground for 5mins on ice using a bench drill fitted with an Eppendorf micropestle. 300 μl DNA extraction buffer was added and centrifuged 13,000 rpm for 1 min; the supernatant was transferred to a fresh 1.5 ml Eppendorf. This process was then repeated. An equal volume of phenol-chloroform-isoamylalcohol (25:24:1) was added, mixed well and centrifuged for 3 min at 10,000 xg. The upper aqueous layer was then transferred to a fresh 1.5 ml Eppendorf. The phenol-chloroform-isoamylalcohol extraction repeated. DNA was precipitated with an equal volume of 70% ethanol and the pellet washed with 3 rounds of 70% ethanol followed by centrifugation (13,500 xg, 5 mins). Finally, the ethanol was removed and the pellet dried at room temperature for Y-X mins before resuspending in 50 μl of sterile deionised H_2_O.

0.5 μl of DNA was added to a 200 μl thin walled PCRtube containing 12.5 μl DreamTaq Green Master Mix (Fermentas, UK), 1 μl forward primer and 1 μl reverse primers (Additional file 1: Table S1), 10 μl DNase-free water. Cycling conditions were 95°C for 5 min followed by 32 cycles (95°C 45s, X°C 45s, 72°C 120s (where X is the annealing temperature for each primer, indicated in (Additional file 1: Table S1)) with a final cycle of 72°C for 10 min. DNA sequences were checked manually, corrected and assembled using Sequencher 4.10.1 (Gene Codes Cooporation, Ann Arbor, Michigan, USA), sequences were assembled using corrected sequences were used to interrogate the NCBI online database using BLAST. The attribution of genus and/or species identity to the isolates was based on the BLAST total score. Total score is not included in the results (Table 1) as it summarises and compares all data from resultant matches for a single query (arbitrary value for each sequence analysed). Instead, Table 1 contains ‘query coverage’ and ‘max ident’ which better describe the ‘quality’ of each final match. In order to be consistent ‘total score’ from the BLAST outputs was used as a means of identification. Where scores were ambiguous, decisions were based on morphology at the light-microscopy level.

**Table 1:** Identification of photosynthetic isolates using DNA barcoding of U16S/U18S rDNA gene

**Isolate**	**Abbr.**	**Division**	**GenBank match ref.**	Coverage%*	Max Ident% **	Max Score ***	**Other (NCBI no)**
Coelastrella saipanensis*	*Cs*	Chlorophyta	AB055800	100	99	3090	BLAST and morphology (JX316760)
*Klebsormidium* sp*.*	*K* sp.	Chlorophyta	FR717537.1	98	99	1929	(JX316761)
*Hantzschia* sp.*	*H* sp.	Bacillariophyta					Morphology
*Chroococcidiopsis thermalis*	*Ct*	Cyanophyta	AB039005.1	100	99	2488	(JX316762)
Microcoleus chthonoplastes*	*Mc*	Cyanophyta	EF654089.1	96	91	1844	Additional file 1: Figure S1
*Mastigocladus laminosus*	*Ml*	Cyanophyta	AB607204.1	95	99	2385	(JX316764)
*Oscillatoria sancta*	*Os*	Cyanophyta	AF132933	96	99	1522	Additional file 1: Figure S2

Sequences compared to the NCBI online database using BLAST. Matches based on ‘Max Score’. Date accessed 17.01.2012.

*% of sequence covered by database hit **% similarity of sequence covered by hit ***The score is calculated from the sum of the match rewards and the mismatch (independently for each segment).

*H*. sp. was identified based on morphology, using images taken from a slide preparation outlined below. An obtained pellet of diatom cells was washed with deionised water twice and centrifuged at 3000 rpm over 10 mins. Supernantant was removed and pellet resuspended in 1ml 30% (w/v) hydrogen peroxide and mixed well. Tube then placed in a water bath at 80-90°C for 1 hr and then allowed to cool. Sample was transferred to 15 ml falcon tube with ~10 ml deionised H_2_O, mixed and centrifuged at 3000 rpm for 3 min. Supernatant then removed and pellet washed twice with distilled water, centrifuging each time and removing the supernatant. Working in a clean flow hood 0.5 ml of mixed suspension was dropped onto coverslips and dried using a hotplate at ~50°C. 1 drop of DPX mounting medium was dropped onto glass slides and using forceps the coverslips with dried diatom were inverted and placed over the drops of DPX. Assembled slides were then dried on the hotplate (at 80°C) overnight. Images were assessed by Dr. S. Spaulding (Colorado State University).

NCBI accession numbers for the Roman Bath isolates are as follows; *Coelastrella saipanensis* JX316760, *Klebsormidium* sp. JX316761, *Hantzschia* sp. JX316762, *Chroococcidiopsis thermalis* JX316763 and *Masticocladus laminosus* JX316764. For the purposes of this paper isolates *Os* and *Mc* have been identified as *Oscillatoria sancta* and *Microcoleus chthonoplastes*. However both sequences contained alignment gaps not acceptable to the NCBI database. Both isolates require re-sequencing for more accurate species identification.

### Staining for lipids

100 μl of cell culture was added to 25 μl DMSO and 1 μl 1% nile red dye in acetone in an eppendorf, mixed well and allowed to stain in the dark for 10 min. This was followed by dilution to 1 ml. Samples were viewed under microscope a Nikon Eclipse 90i and a confocal D-Eclipse C1. Nile red produces strong orange fluorescence at 543 nm/598 nm (excitation/emission) and BODIPY produces a strong green fluorescence at 493 nm/503 nm.

### Oil extraction and transesterification

Total lipids were extracted from samples of algal biomass essentially as described by Bligh and Dyer (1959). Lyophilised algal samples were weighed and placed in MP Biomedical ‘lysing matrix E’ (Cambridge, CB1 1BH, UK) tubes together with 1.5 ml sterile deionised water. Samples were beaten in a Fastprep FP120 beadbeater (Thermo Scientific Savant, Surrey, UK) at setting 6.5 at intervals of 45 seconds for a total of 3 minutes. Tube contents were removed via a pipette and rinsed with a CHCl_3_:MeOH (2:1) mixture. For each sample ~5 ml CHCl_3_:MeOH (2:1) was added and the sample vortexed. Samples were then centrifuged at 3000 rpm for 5 min. The bottom layer was removed to a round bottom flask. Two subsequent rinses were performed to remove the remaining oils by adding 1.5 ml CHCl_3_ to the sample, vortexing and centrifuging. Giving a total sample volume of 10 ml.

To transesterify the samples, MeOH (10 ml) and 18M H_2_SO_4_ (1 ml) were added to the sample, which was then held at reflux for 4 hours. The crude mixture was washed twice with H_2_O (50 ml) to remove the acid and resulting glycerol. The organic layer was isolated, the solvent was removed under vacuum and the samples were dried and dissolved in 2 ml of 1,4-dioxane (99 +%) and analysed by an Agilent 7890A Gas Chromatograph with capillary column (60m × 0.250mm internal diameter) coated with DB-23 ([50%-cyanpropyl]-methylpolysiloxane stationary phase (0.25m film thickness) and a He mobile phase (flow rate: 1.2ml/min) coupled with an Agilent 5975C inert MSD with Triple Axis Detector. The column was pre-heated to 150°C, the temperature held for 5 minutes and then heated to 250°C (rate of 4°C/min, then held for 2 min). The samples were quantified by comparison to known standards purchased from Sigma Aldrich.

## Results

### Observations, water analysis and identification of isolated photosynthetic microorganisms

The Roman Baths appear to have extensive microbial mat communities submerged beneath the water surface, some with striking blue-green patches. There are also filamentous ‘hairy’ brown mats that grow around evolved gas bubbles. These eventually create gelatinous balloons-like structures that rise to the surface, anchored to the microbial mat below by filamentous ‘ropes’. A bright orange muddy residue, possibly caused by bacteria, detritus and iron hydroxide precipitates form a thick layer (~2-3 cm), between the mats and stone surfaces. In addition to iron, the spring water contains high concentrations of calcium, sulphate, sodium and chloride (Kellaway, 1991). There are also visible mineral deposits, which form dependant on water agitation, cooling and oxygenation (Kellaway, 1991). Water analyses of the Great Bath (GB) and Kings Bath (KB) show that the abiotic conditions in the baths have remained stable since records began in 1874 (see Additional file 1: Table S2, for full water analysis). The temperature of the Great Bath and Kings Bath were 39.0°C and 45.0°C respectively, measured 30cm below the surface.

**Figure 1:**
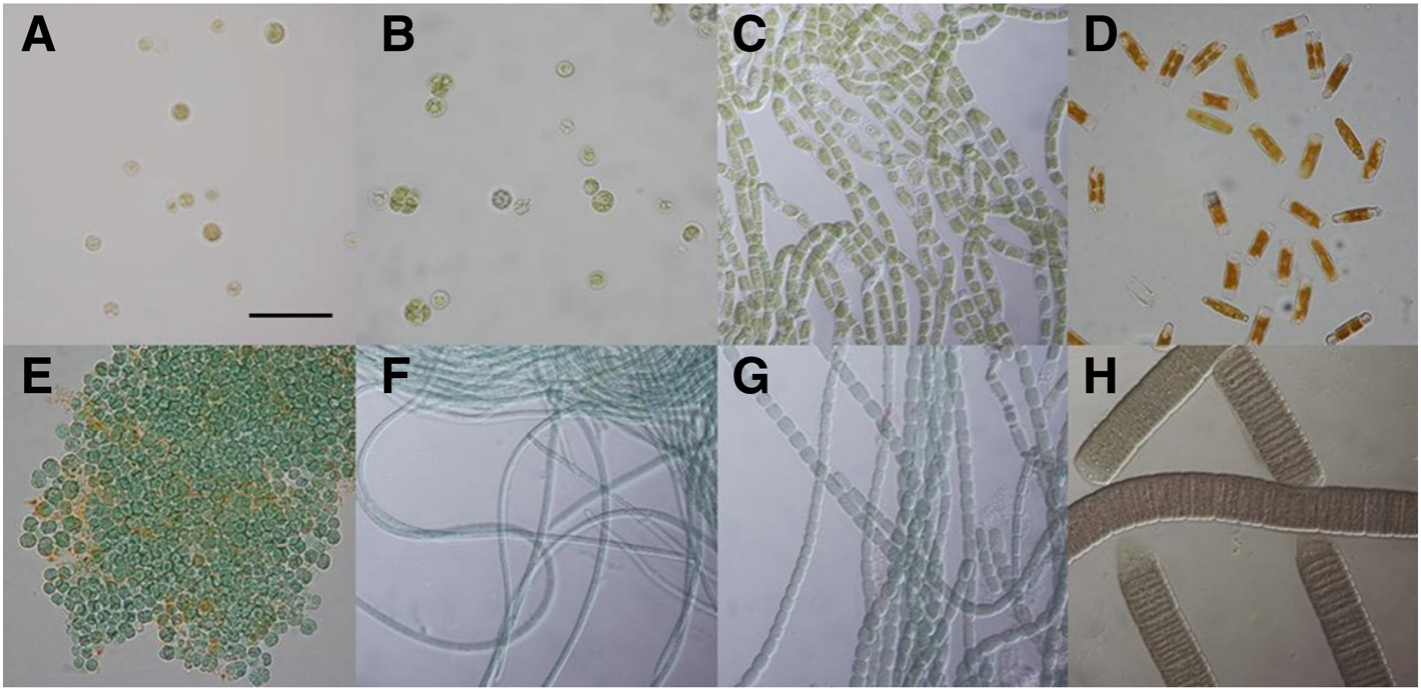
**Light microscope images of algae isolated from the Roman Baths (x60).** All algae were isolated from the Great Bath unless indicated (* = isolated from Kings Bath). **A**, *Chlorella emersonii* (*Ce*); **B**, *Coelastrella saipanensis* (*Cs*); **C**, *Klebsormidium* sp. (*K* sp.); **D**, *Hantzschia* sp. (*H* sp.); **E**, *Chroococcidiopsis thermalis* (*Ct*); F*, *Microcoleus chthonoplastes* (*Mc*); G*, *Mastigocladus laminosus* (*Ml*); H*, *Oscillatoria sancta* (*Os*). Bar = 20 μm.

In Figure 1, images of the isolates from the Roman Baths, alongside the reference alga *Chlorella emersonii* are shown. These are *Chlorella emersonii* (A), two green algae (B, C), a diatom (D) and four cyanobacteria (E, F, G, H). Isolates were identified as follows and will be referred to in figures in the same order by their abbreviations in brackets; B: *Coelastrella saipanensis* (*Cs*), C: *Klebsormidium* sp. (*K* sp.), D: *Hantzschia* sp. (*H* sp.), E: *Chroococcidiopsis thermalis* (*Ct*), F: *Microcoleus chthonoplastes* (*Mc*), G: *Mastigocladus laminosus* (*Ml*) and H: *Oscillatoria sancta* (*Os*). It should also be noted that there appears to be an abundance of microalgal species present in the baths, more than are discussed in this paper. Filamentous cyanobacterial species (*O.sancta, M.chthonoplastes, M.laminosus*) were isolated by from sample scrapings of microbial mats and contaminants removed using the ‘agar plate scoring method’. All other species were isolated using the ‘filter paper method’.

Microalgae are often ‘elastic’ in their ability to alter their morphology based on their growth conditions. In a unique environment such as the baths, algae may potentially look different to species standards. In addition, it is not uncommon for algae which are distantly related to look similar and vice versa. As such identifying algae based on morphology is very difficult, therefore DNA barcoding was the preferred method for identification of isolated photosynthetic microorganisms. Isolates were assigned species or genus level identification using Basic Local Alignment Search Tool (BLAST) using the ‘total score’ values and in some cases images from online culture collections to confirm matches (see methods). In order to be consistent ‘Total score’ from the BLAST outputs was used as a means of identification. Any isolates with multiple hits of the same score, visual identification was used to help confirm an ID.

The isolate identified in Table 1 as *C. saipanensis*, initially received identical BLAST scores for *Coelastrella saipanensis* and *Ettlia texensis*. Upon closer examination both entries in the database comprised of identical nucleotide sequences. Despite the difficulty in identifying algae based on morphology alone, images from online culture collections were used in order to identify this isolate from the two identical database ‘hits’. Images of *Coelastrella* sp. in online culture collections showed similar cell granularity (‘speckled’ inclusions) to *C. saipanensis* but did not show aggregates of diving cells inside mother cells (a feature of *Ettelia* spp.). Therefore has been identified as *C.saipanensis*. For *K*. sp. there were many similarly scored species of the genera *Klebsormidium*. However, there were no distinct physical features present to identify a species based on morphology.

Identifying *H.* sp. to the species level proved problematic. There were many very similarly scored matches from different groups of diatoms that are morphologically very distinct. Diatoms, unlike many unicellular algal groups, can be more easily identified using morphology alone to the expert eye. Identification of *H*. sp. was subsequently based on images from a slide preparation (described in methodology), which were subsequently assessed by Dr. S. Spaulding (Colorado State University) for identification. For *M. chthonoplastes* most of the outputs in the BLAST search regardless of sorting had a ~98% query coverage from uncultured clones and *Microcoleus chthonoplastes* strains (with max idents of 91%). However, *Geitlerinema* cf. *acuminatum,* had 77% query coverage (percentage of the input sequence covered by individual sequences in the database) but a 97% max ident score (maximum percentage of high scoring pairs in a segment). Little has been published on *Geitlerinema* spp. but a few culture collection images do more closely match the growth morphologies of the isolate (*Mc*) than *Microcoleus* i.e. *M. chthonoplastes* isolate grows as an amorphous tangle of filaments like *Geitlerinema* as opposed to discrete bundles of fibres like *Microcoleus* spp.

Roman Bath isolate sequences for *Oscillatoria sancta* and *Microcoleus chthonoplastes* were not accepted to the NCBI database due to alignment gaps and some chimeric sequences. This may be attributed to their very thick gelatinous outer cell walls which likely contain some contaminant bacteria. Both isolates require re-sequencing for more accurate species identification.

### Temperature tolerance

Results of the temperature tolerance experiments (Additional file 2: Table 2) show that the eukaryotic algae (*C.emersonii*, *C.saipanensis*, *Klebsormidium* sp. and *Hantzschia* sp.) behave similarly and are able to grow at 30°C but do prefer lower temperatures. The cyanobacterial isolates have more variation between them but all show good growth at 35°C and some growth at 40°C, unlike the eukaryotes. Based on these results further growth experiments were carried out at 20°C, 30°C and 40°C, to give a low, medium and high value for comparison.

During isolation and culture maintenance of the Roman isolates it was noted that *O. sancta, C.thermalis* and *H.* sp. showed sensitivity to the light intensity used to culture stocks of *C.emersonii* (200 μMol m^-2^ s^-1^) (data not shown). For this reason, it was decided to use a ‘moderate’ light intensity (80 μMol m^-2^ s^-1^) and shaking speed for temperature tolerance experiments. Although this allows for results to be comparable for a specific set of culture conditions, the culture parameters are likely to have been suboptimal for a number of the species examined (e.g. *C.emersonii* potentially extending to the other green algae *K*.sp, *C.saipanensis* and even the cyanobacterial isolates). Growth media may also be suboptimal.

**Figure 2:**
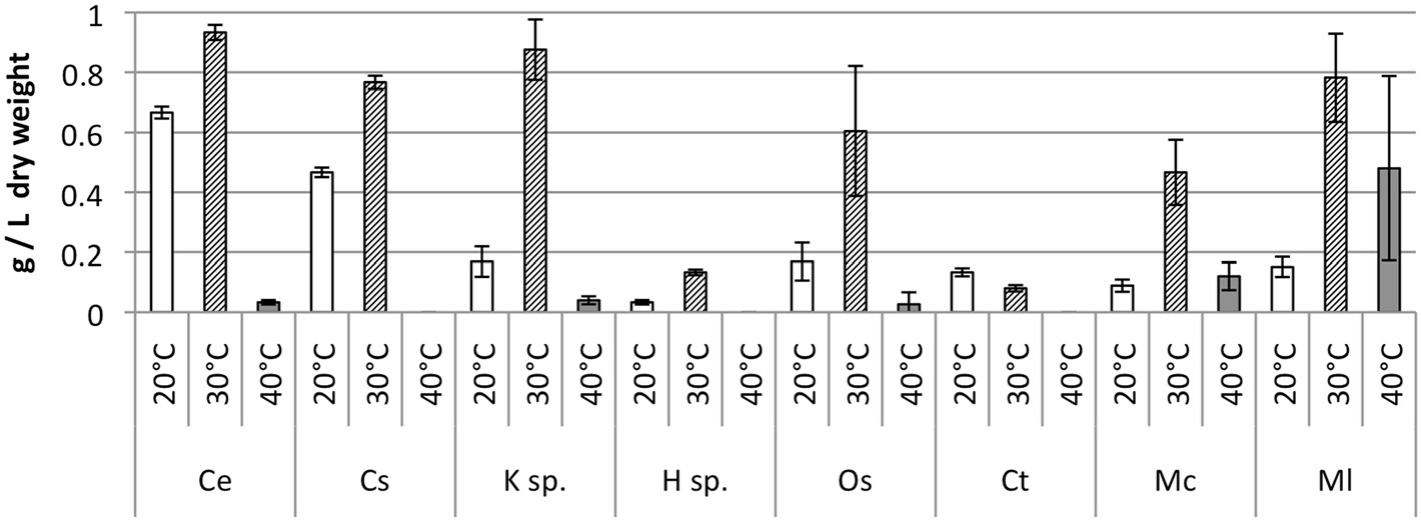
**Growth of *Ce *and Roman Bath isolates in nitrogen sufficient medium after 12 days, measured as dry weight (g l**^**-1**^**).**

To further examine the growth rate under nitrogen enriched conditions the microalgae were cultured over 12 days and sampled periodically. To clearly communicate this information the final dry weights are presented in Figure 2, all other data is presented in full in the Additional file 1: Figures S3-S5. These experiments supported the preliminary data, where the eukaryotic algae all accumulated biomass at the lower temperature of 20°C, were more productive at 30°C but generally did not grow at 40°C. *K*. sp. and *C. saipanensis* accumulated the most biomass at 30°C, comparable to the reference alga *C. emersonii*. Within the cyanobacteria, the single celled species *C. thermalis* showed better growth at lower temperatures. *M. chthonoplastes*, *M. laminosus* and *O. sancta* acquired the most biomass at 30°C, with *M. chthonoplastes* and *M.laminosus* showing better growth at 40°C than 20°C. This was not found to be the case for *O.sancta* whose growth was impaired at 40°C. *M. laminosus* acquired the most biomass of all the cyanobacteria, at a temperature of 30°C.

Of all the algae tested, *C. emersonii* accumulated the most biomass at 20°C, *K* sp. the most at 30°C and *M.laminosus* the most biomass at 40°C. Both *H.* sp. and *C. thermalis* were not sufficiently productive at any of the temperatures investigated, achieving <0.2 g l^-1^ after 12 days, unlike other species which achieved between 0.5-0.9 g l^-1^ after 12 days.

### Cytochemical quantitation of neutral lipid content

**Figure 3:**
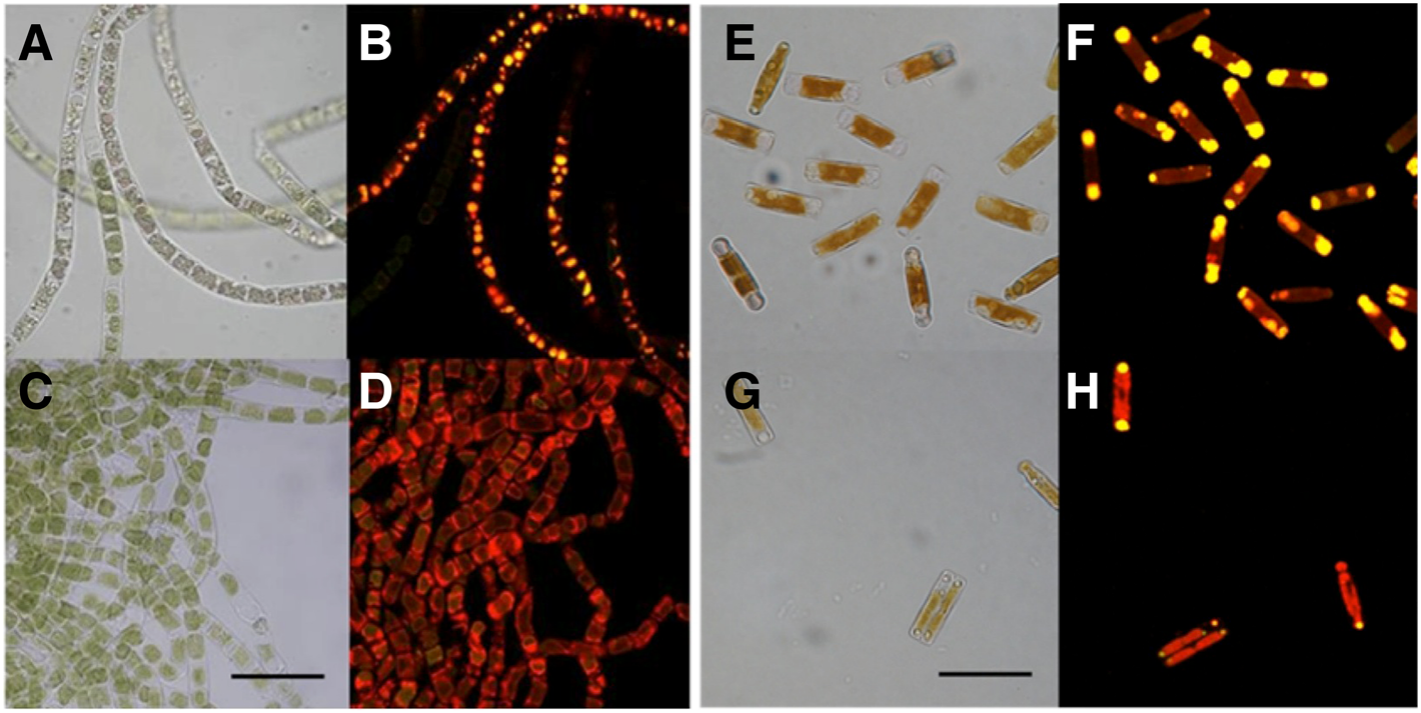
**Microscope images of *Klebsormidium *sp. cultivated at 20°C (A,B) and 30°C (C,D) and starved of nitrogen.** Microscope images of *Hantzschia* sp. cultivated at 30°C with sufficient nitrogen (**E,F**) and starved of nitrogen (**G,H**). Stained with nile red. Bar = 20 μm.

Staining gave a visual indication of the neutral lipid products contained in a sample (Figure 3). Nile red stains lipids and fluoresces red, where the lipids are predominantly unsaturated and yellow where the lipids are saturated. Photosynthetic pigments including chlorophyll present in eukaryotic algae and cyanobacteria auto-fluoresce at the same excitation wavelengths as nile red. This can make it difficult to identify stained lipids against the autofluorescent ‘backdrop’ of the cell. However the fluorescence of these pigments is useful in assessing the health of the cell as stressed cells undergo breakdown of these pigments. This can be seen in Figure 3A and B showing nitrogen starvation of *Klebsormidium* sp. at 20°C. At 20°C *K*. sp. (Figure 3A and B) has clearly visible lipid droplets and a noticeable lack of chlorophyll. At 30°C however, during nitrogen starvation, *K* sp. shows little or no breakdown in chlorophyll or accumulation of lipids (Figure 3C and D).

Nitrogen starvation usually encourages accumulation of lipids in most microalgae (Cha et al. 2011). However, *H.* sp. at 30°C shows higher accumulation of lipids under nitrogen sufficient conditions (Figure 3E and F) compared to nitrogen starved conditions (Figure 3G and H). Staining images for all species are presented in the Additional file 1: Tables S3-S5, in full.

### Lipid yield

To assess the effectiveness of the microalgae to produce biodiesel, the neutral lipids from the microbial samples (the weight of which is given in Figure 2) were isolated, using a chloroform and methanol solvent extraction and then converted into FAME through acid-catalysed transesterification and analysed by GC-MS.

**Figure 4:**
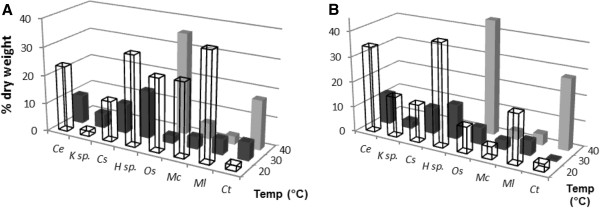
FAME produced from the Roman Bath isolates as a percentage of dry weight grown under A) nitrogen enriched and B) nitrogen starved conditions.

Reducing the nitrogen content in the culture medium for green algae generally increases the neutral lipid accumulation in the cell (Cha et al. 2011). This effect is illustrated by the reference species in *Chlorella emersonii* where the FAME produced from the sample increased from 23 wt% to 34 wt% of the dry weight under nitrogen starved conditions at 20°C (Figure 4). A similar effect was seen with *K*. sp. (increasing from 1.9wt% to 16wt%) and *H.* sp. (from 31.9 to 40.5%). In contrast nitrogen-depletion did not change the amount of FAME recovered dramatically for *C. saipanensis*. Irrespective of any other effect, the green algae produced less neutral lipid at higher temperatures. Nitrogen-starvation of cyanobacteria had a detrimental effect on the lipid percentage at both 20°C and 30°C. At 40°C, however, a high percentage of FAME (10.4-45.6%) was recovered from *O. sancta* under most conditions (with the exception of 30°C). This effect was not observed in *M. laminosus*, *M. chthonoplastes* or *C. thermalis*.

**Figure 5:**
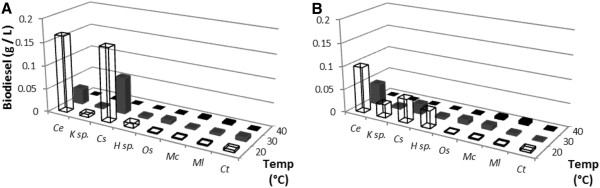
Total amount of biodiesel recovered from the Roman Bath isolates grown under A) nitrogen enriched conditions and B) nitrogen starved conditions.

**Figure 6:**
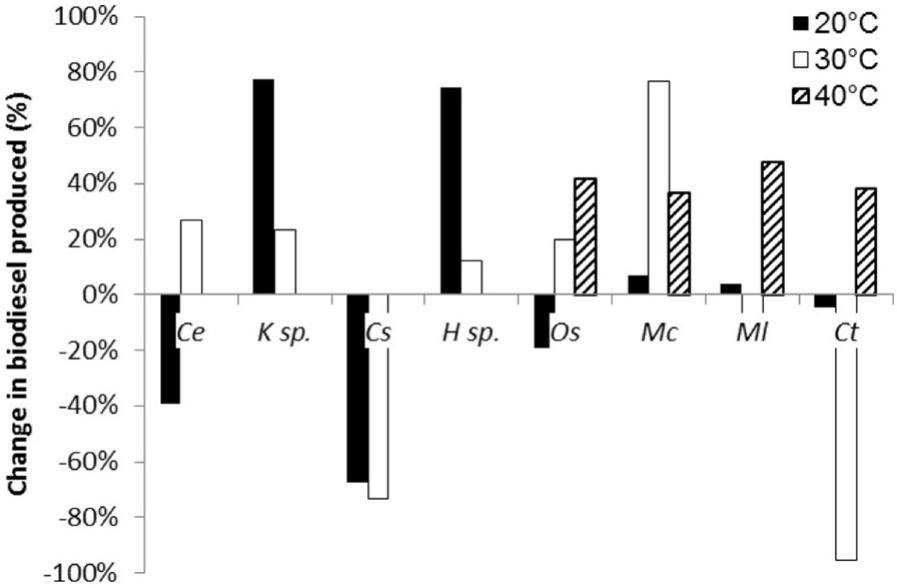
% change in the total amount of biodiesel produced on nitrogen starvation of the algal species.

Reducing the nitrogen content in the growth media may have a detrimental effect on growth rate. Where this is the case, oleaginous microbes will fail to produce a substantial amount of biofuel on a per unit biomass basis (Figures 5 and 6). The highest biodiesel production was observed for *C. emersonii* and *C. saipanensis* at 20°C under nitrogen enriched conditions, where the lower content of glyceride lipids within the biomass were compensated by a higher growth rate. *K*. sp. and *H.* sp. biomass increased in the glyceride content when nitrogen content was reduced. None of the cyanobacteria produced a large amount of biodiesel irrespective of nitrogen conditions at 20°C and 30°C (Figure 5) compared to green algae *C. emersonii* and *C. saipanensis* (>0.15 g l^-1^ of biodiesel). For the most part, this was due to low levels of biomass accumulation in the cyanobacterial isolates (Figure 2). However, at 40°C, nitrogen reduction resulted in recovery of a larger quantity of biodiesel from the cyanobacteria, though this represented only a small amount of biodiesel overall.

### Biodiesel profile

**Figure 7:**
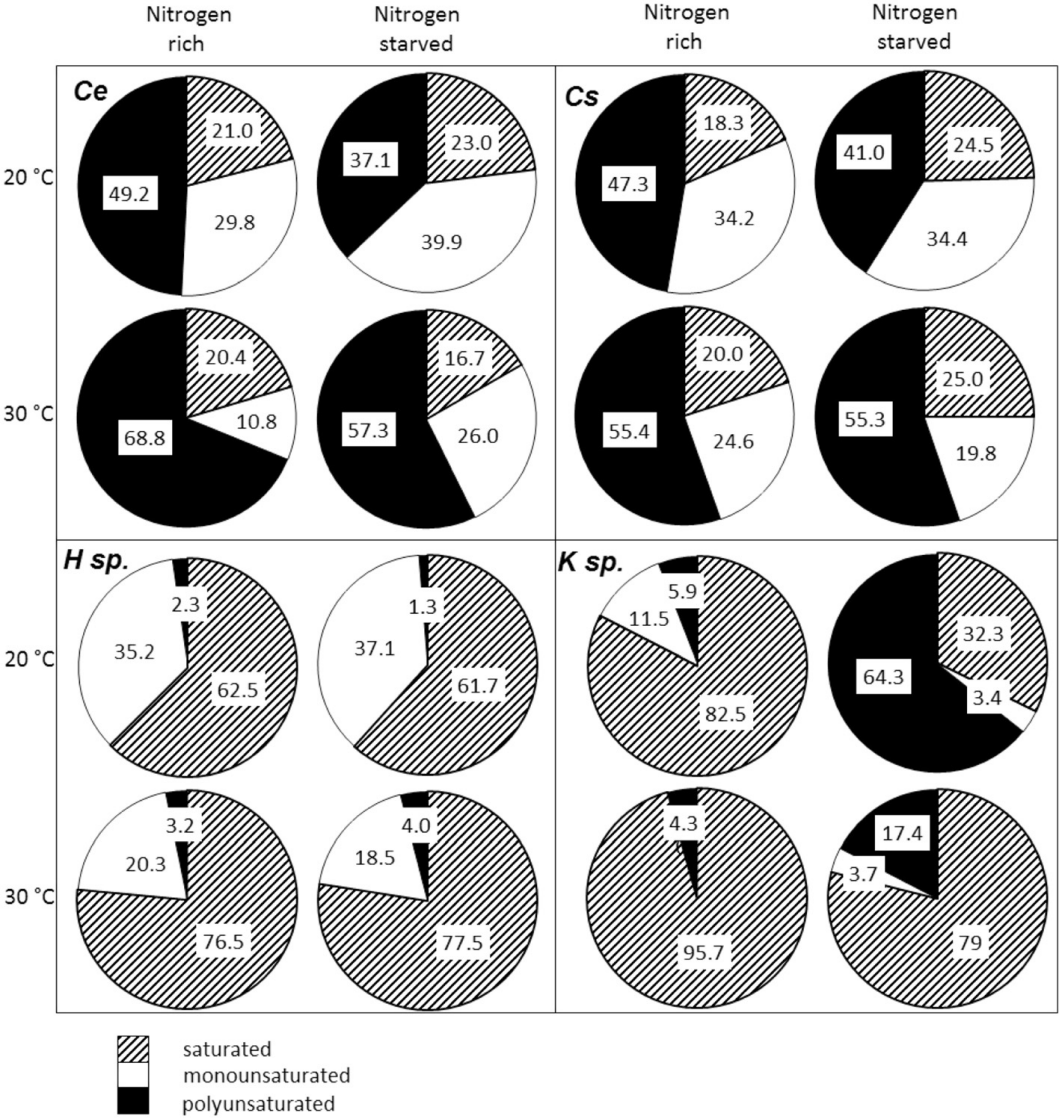
FAME profile of the microalgae cultured throughout this investigation.

**Figure 8:**
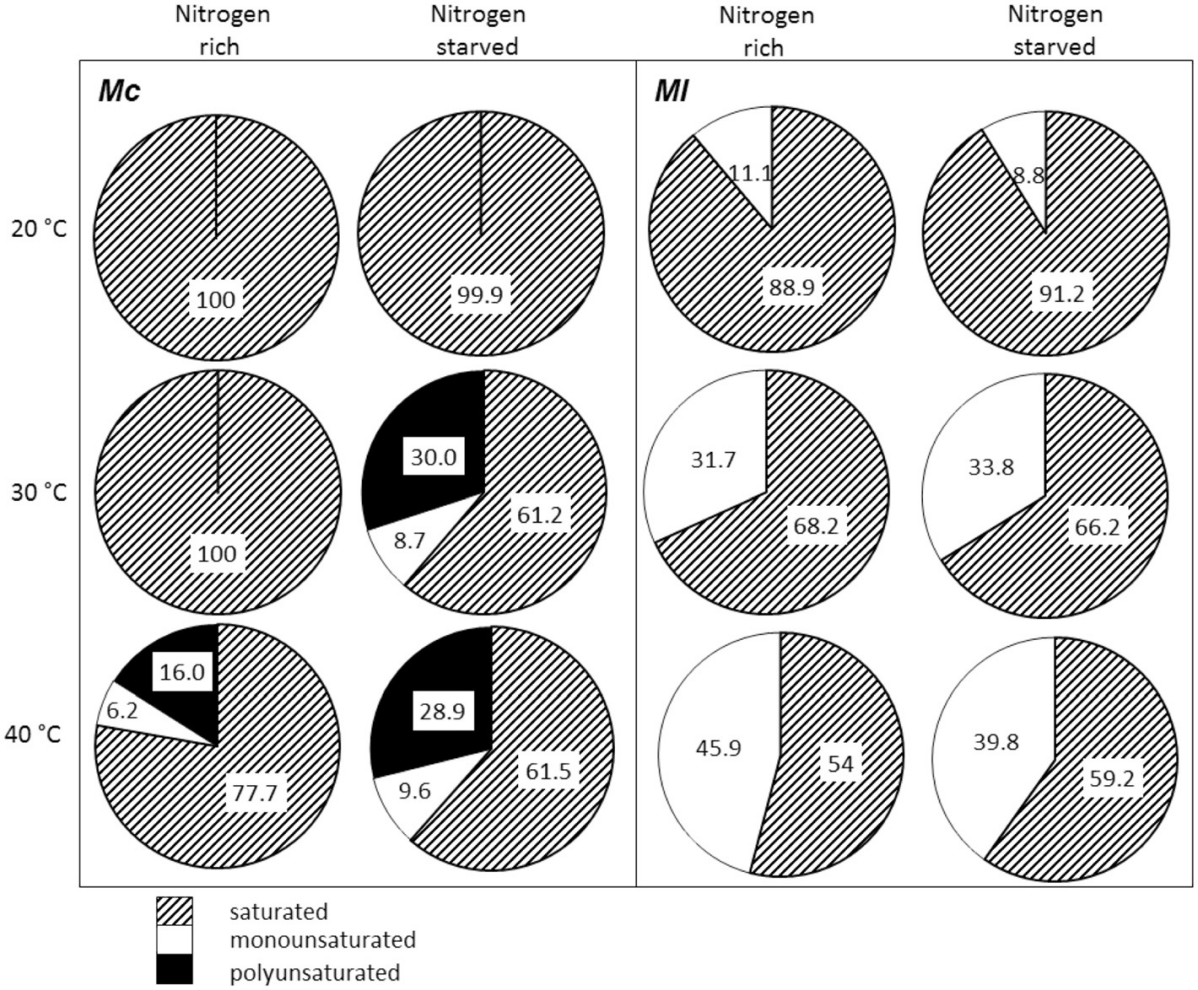
FAME profile of the cyanobacteria MC and ML.

The properties of biodiesel are highly reliant on the FAME profile (Fortman et al. 2008). Although chain length and the degree of unsaturation do have an effect on the fuel properties, the FAMEs can be placed into three loose categories to predict the fuel performance; saturated, monounsaturated and polyunsaturated esters (Figures 7 and 8). Biodiesel fuels rich in saturated esters have a superior cetane number but poor viscosity and low temperature properties; monounsaturated esters have acceptable low temperature properties and viscosity; fuels high in polyunsaturates have excellent low temperature properties and a low viscosity but a poorer cetane number and oxidative stability. Full FAME profiles were obtained for the isolated algae and are given in the Additional file 1: Tables S6-S8.

On reduction of the nitrogen content during the culturing of *C. emersonii* the FAME profile did not change dramatically. However, an increase in temperature, in both nitrogen regimes, resulted in an increased polyunsaturated component predominantly at the cost of the monounsaturated fraction (Figure 7). A similar outcome was observed for *C. saipanensis*. In contrast to *C. emersonii* and *C. saipanensis* nitrogen starvation of *K*. sp. increased the production of unsaturated FAME whereas both the nitrogen enriched 20°C and 30°C samples contained predominantly saturated FAMEs. Like the green algae *C. saipanensis* and *C. emersonii*, the FAME profile of *H.* sp. did not change significantly following nitrogen starvation. However, raising the growth temperature (20°C – 30°C - 40°C) increased the proportion of the saturated component. The relatively high level of saturates recovered form both *K*. sp. and *H.* sp. would result in a poor quality biodiesel.

The FAME profiles of the cyanobacteria were much simpler than those of the green algae. Unfortunately, insufficient biodiesel was recovered from *O. sancta* or *C. thermalis* to enable complete analysis of the FAME profile (see Additional file 1). *M. chthonoplastes* produced only saturated esters at both low temperatures and in nitrogen-rich conditions at 30°C (Figure 8). Nitrogen depletion, or an increase in temperature to 40°C, resulted in an increase in unsaturated esters and composition more suitable for biofuel. *M. laminosus* produced no polyunsaturated esters under any of the conditions examined. At low temperatures there were around 90% saturates which decreased to approximately 66% when the temperature was increased to 30°C, and to approximately 50% at 40°C. Nitrogen-starvation had little effect on the FAME profile at any temperature.

## Discussion

### Isolation and identification of microalgae

The excavation of the Roman Baths to uncover the present buildings and associated water-filled baths began in 1878 (Byrne 2008). Consequently, the opportunity for the colonisation of the baths by microalgae and other microbes has existed for some 134 years. Despite the city-centre location of the Baths and the relatively high water temperature initial sampling in the baths revealed the presence of multiple microalgal species. The isolates *O. sancta*, *M. chthonoplastes*, *M. laminosus* and *H*. sp., proved the predominant species and were repeatedly found when attempting to isolate other species. Whilst the present work isolated and identified 7 species, our efforts were not exhaustive and further species remain for future isolation.

The first step to identifying algae present in the baths was to establish axenic unialgal cultures. Whilst other algae are seldom difficult to remove, bacteria are often physically associated with the muciferous layer of the algal cell walls and are not always eliminated by standard serial dilution and plating techniques. Impurities in agar (derived from algae) can also prove inhibitory to the growth of some algae and may encourage growth of bacterial and fungal contaminants. Some cyanobacterial isolates grew poorly on agar most likely due to toxic component in the agar (Dworkin and Falkow, 2006). Micromanipulation, filtration and use of chemicals have been successful with some groups (Ferris and Hirsch, 1991). The use of antibiotics appears attractive but must be used in a narrow concentration range to avoid damage to the chloroplast in eukaryotes and lethality to cyanobacteria (Issa, 1999).

In order to amplify and sequence 16S (cyanobacterial) or 18S (eukaryotic) rDNA gene sequencing, the isolates needed to be subjected to some decontamination. Filamentous cyanobacterial species (*O.sancta, M.chthonoplastes, M.laminosus*) were isolated by from sample scrapings of microbial mats and contaminants removed using the ‘agar plate scoring method’. All other species were isolated using the ‘filter paper method’. However, it is evident that the microbial mat communities present in the Roman Baths are required for microbial growth and survival. Hence it is also highly likely that resident algae have formed relationships with other organisms present (Croft et al. 2006). Although most of the contaminants have been removed, the isolates are described as unialgal.

Employing a DNA-barcoding approach utilising the U16S and U18S rDNA gene sequences and a NCBI BLAST search as an identification tool proved problematical for *M.chthonoplastes*, *C.saipanensis*, *Hantzschia* sp. and *Klebsormidium* sp. This appears to arise due to both a relative paucity of gene sequences from microalgal species and the presence of incorrectly assigned sequences due to poor regulation of the NCBI database. Naturally, there is also a substantial bias toward more commonly used species. There is still uncertainty about the optimum fragments of DNA to use for identification (Surek, 2008). *Cox1* is the standard for most ‘higher animals’ but is not suitable for green algae and higher plants because its rate of evolution is too slow (Surek, 2008). Common gene targets for algal identification are rRNA genes, mitochondrial genes and plastid genes, but each may fail to provide a conclusive result. Clearly there is a need to identify a single universal short DNA fragment that gives a clear identification of species (Surek, 2008).

Differences in geographical location can result in changes in target sequences *within* a species. This has been demonstrated for *M. chthonoplastes* (Garcia-Pichel et al. 1996) and *Synechococcus* sp. (Miller and Castenholz, 2000). Whilst this type of analysis has provided evidence that more thermotolerant lineages evolved from less thermotolerant ancestors (Miller and Castenholz, 2000), such ecologically induced changes can further complicate DNA sequence-based species identification.

Where DNA-barcoding proved inconclusive, algae were subjected to morphological examination at the light microscope level and images compared to online culture collections (e.g. the Culture Collection of Autotrophic Organisms (CCALA)). This approach proved successful for *C.saipanensis* but not for *H*.sp. Microalgae often lack distinct morphological features that can make them hard to identify (Pulz and Gross, 2004). Moreover, algal development can be plastic with cells changing shape and size during the lifecycle and in response to changes in culture conditions (Cheng et al. 2011). Consequently, algal identification and taxonomy demands a ‘polyphasic’ approach that uses both molecular and morphological data as employed in this study (Surek, 2008).

### Thermotolerance

Cyanobacteria are often found in warm, low nutrient environments (De Winder et al. 1990), and are typically more thermotolerant than eukaryotic algae (Barsanti and Gualtieri, 2006). Members of the single-celled cyanobacterial genus *Thermosynechococcus* are capable of surviving at 73-74°C, whilst thermophillic filamentous cyanobacteria typically occupy a lower temperature range of 55-62°C (Seckbach, 2007). In accord with this the present study found 50% of the isolates from the Roman Baths were cyanobacteria, and that these species remained viable and productive at higher temperatures than the eukayotic isolates.

Eukaryotic algae are generally absent from environments above 56-60°C largely due to the instability of organellar membranes at high temperatures (Tansey and Brock, 1972). Members of the Rhodophyta such as *C. caldarium* have been found to withstand 57°C (Seckbach, 2007). However, none of the Roman Bath isolates were from this genus. The comparatively poor growth of the eukaryotic isolates compared to the cyanobacteria, suggests that they were present in cooler locations within the baths i.e. on exposed surfaces close to the waterline*.*

In some cases there was a lack of correlation between growth rates at high temperatures *in vitro* and the prevalence of a particular species in the Baths. For example, *O. sancta* showed limited growth at 40°C (Figure 2) despite being the dominant microalgal species in the baths where it forms filamentous ‘balloons’ around evolved gas bubbles. In both environments the species is brown in colour rather than the typical blue-green. This is consistent with previous studies showing that high light or high temperature result in *Oscillatoria* sp*.* making a similar colour change and an associated reduction in growth rate (Gribovskaya et al. 2007). To ensure comparability of temperature effects all other conditions were *moderated* in order to be consistent for all species tested. For example, *C. emersonii* exhibits a faster growth rate at a higher light intensity over longer light: dark cycles, however during culturing these conditions were found to be detrimental to growth rate for *C. thermalis*, *H*. sp. and *O. sancta* (data not shown). Growth was therefore highly likely to be suboptimal for all the species investigated and further work is required to determine optimal growth conditions.

### The extreme conditions in the baths favour microbial communities

The poor performance of unialgal cultures may reflect a requirement for cooperation in a microbial community. Microbial mats are important primary producers in extreme habitats where they provide environments that facilitate survival (Pattanaik et al. 2008). Evidence also suggests that cultured communities are more productive than monocultures (Croft et al. 2005). Microbial mats are prevalent in the Roman Baths and may therefore enable species to survive in this high temperature environment. The Roman Baths contain good levels of most trace elements required to support growth. However, some of these nutrients are in large excess which in turn can cause stress to algal cells. Calcium, sodium and chloride levels are much higher than standard media used for culturing these groups of algae (Barsanti and Gualtieri, 2006) and iron levels are much higher in the baths than most freshwater sources (Kellaway, 1991). In many aquatic environments iron is a limiting nutrient for productivity, yet in the baths it is at a similar concentration to most growth media. However it is important to note that for some species this could represent an excess. High iron concentrations have a negative effect on phytoplankton growth and increase oxidative stress (Estevez et al. 2001). Silicon is also plentiful in the bath, which presumably accounts for the abundance of *H.* sp.

### Effect of culture conditions on FAME production and composition

Although nitrogen starvation and other stresses trigger neutral lipid accumulation in eukaryotic microalgae this is generally associated with a reduction in growth rate (Illman et al. 2000). This reduction was observed for *C. saipanensis* (Figure 2), where more FAME was produced under nitrogen starvation on a per cell basis (from 14.3 to 14.9 wt%) but had a low total productivity of biodiesel due to limited biomass production under these conditions (Figure 5). This trade off is therefore an important consideration when selecting microalgae for a particular production regime. Interestingly, although the cyanobacteria examined in this work accumulated a lower diversity of FAMEs than the eukaryotic algae, these species produced high levels of neutral lipids that were converted into FAME (up to 45 wt%) under nitrogen-rich conditions (Figure 5). The fact that cyanobacteria can accumulate lipids in the thylakoid membrane under conditions promoting photosynthesis and high growth rates may explain this behaviour (Karatay and Dönmez 2011).

The green algae *C. saipanensis* produced a large amount of polyunsaturated esters under all the conditions trialled similar only to the reference algae *C. emersonii* (refer to data). This FAME profile is roughly equivalent to sunflower or soybean oil (Knothe et al. 2005) and the resulting biodiesel would therefore have similar fuel properties including relatively high oxidative instability. Biodiesel produced from *H.* sp. and *K*. sp. was more saturated than that obtained from the other microalgal species (Figure 7). This effect increased with rising temperature (for *H*. sp. and *K.* sp. and increase in temperature from 20-30°C increased saturates by 14.0% and 13.2% respectively). Fuels high in saturates tend to have high cloud points, poor low temperature behaviour and a high viscosity (Knothe et al. 2005). Consequently, biodiesel produced from *H.* sp. and *K*. sp. would be unsuitable for high blend levels but would have combustion qualities akin to high performance diesel fuel such as a high cetane number, lower NO_x_ emissions and be highly oxidatively stable.

The cyanobacteria *M. chthonoplastes* and *M. laminosus* were rich in saturated esters (Refer to data). Although difficult to generalise, cyanobacteria tend to produce saturates in larger quantities than other species especially when cultivated at higher temperatures (Balogi et al. 2005; Nanjo et al. 2010; Dinamarca et al. 2011). Since unsaturated esters are more oxidatively stable this may be a direct response to a high temperature environment. Interestingly, the levels of saturated esters isolated from *M. laminosus* were reduced when cultured at higher temperature and at 40°C the biodiesel produced was almost 50% monounsaturated. Monounsaturated esters on balance have the most promising fuel properties, and biodiesel rich in these esters, such as rapeseed methyl ester or olive oil methyl ester, can be used at higher blend levels than other types of biodiesel so with careful control of the culture conditions biodiesel with suitable physical properties can be produced from the algal species isolated.

Our aim was to identify thermotolerant, oleaginous microalgae with potential as a source of renewable biodiesel. The Roman Baths proved to support a rich diversity of microalgal and cyanobacterial species. A total of 3 green algae, 1 diatom and 4 cyanobacteria were successfully established as unialgal cultures, and the majority assigned a species identification using a DNA barcoding approach. Whilst, a number of species produced high levels of neutral lipids, suitable for FAME production, under a range of conditions, all were more productive at lower temperatures than found in the Baths. Whilst these species do not sustain high productivity at extreme temperatures, an ability to survive temperature spikes in an open pond production system is an extremely desirable trait (Pulz and Gross, 2004). To date, the few species that are successfully cultivated commercially in open ponds are extremophiles able to grow in a highly selective environment (Xu et al. 2009). This work highlights the diversity in form, products and behaviour of algal species isolated from the same extreme environment and the importance of screening for new species. However a rapid species screening method requires a quick and efficient extraction method, which does not affect FAME profiles and ideally is scalable.

The culture conditions used to screen the microalgae were not optimised, suggesting that some of species could be developed into effective biodiesel producers. Four species, *K.* sp*., M. chthonoplastes, M. laminosus and O. sancta*, are filamentous, which could reduce harvesting and dewatering costs. One of these, *M. laminosus*, is also nitrogen-fixing species, which could again reduce input costs. Both these features could assist process on scale-up.

## Competing interests

The authors declare that they have no competing interests.

## Supplementary Material

Additional file 1 **Bioprospecting the thermal waters of the Roman Baths: Isolation of oleaginous species and analysis of the FAME profile for biodiesel production. Table S1**. Primer pairs used for amplification of either U16S (cyanobacteria) or U18S (eukaryotic algae) gene regions. Second primer sets created if sequences were too short (i.e. <1000bp). * modified from (Taton et al. 2003), **(Cuvelier et al. 2008). **Table S2**. Composition of bath thermal waters (Great Bath (GB) and Kings Bath (KB)) compared to historical measurements from the kings spring (Kellaway 1991). Analysis performed by Severn Trent Services, unless stated all data in mg/L. **Table S3**. Confocal microscope images of *C.emersonii* and roman bath isolates stained with nile red after cultivation at 20°C temperatures with and without nitrogen starvation. **Table S4**. Confocal microscope images of *C.emersonii* and roman bath isolates stained with nile red after cultivation at 30°C temperatures with and without nitrogen starvation. **Table S5**. Confocal microscope images of *C.emersonii* and roman bath isolates stained with nile red after cultivation at 40°C temperatures with and without nitrogen starvation. **Figure S1**. *Microcoleus chthonoplastes* full 16S rDNA sequence. **Figure S2**. *Oscillatoria sancta* full 16S rDNA sequence. **Table S6**. FAME% profiles of the microbes cultured at 20°C. **Table S7**. FAME profiles (%) of the microbes cultured at 30°C. **Table S8**. FAME profiles (%) of the microbes grown at 40°C, rows given in italics are partial FAME profiles based on the limited amount of material that was available. **Figure S3**. Dry weight of the isolates grown under nitrogen enriched conditions at 20°C. **Figure S4**. Dry weight of the isolates grown under nitrogen enriched conditions at 30°C. **Figure S5**. Dry weight of the isolates grown under nitrogen enriched conditions at 40°C. Click here for file

Additional file 2: Table 2 Temperature tolerance experiments comparing growth of *C.emersonii* and Roman Bath isolates. Growth was assessed visually by comparing samples across the temperature ranges. – no growth, + poor growth, ++ good growth, +++ vigorous growth, NT not tested. Click here for file
