# Supplementary material for: Bioprospecting the thermal waters of the Roman baths: isolation of oleaginous species and analysis of the FAME profile for biodiesel production
Source: AMB Express. 2013 Jan 31;3:9. doi: 10.1186/2191-0855-3-9 (PMC3571919; doi:10.1186/2191-0855-3-9)
Supplement: Additional file 1 — Bioprospecting the thermal waters of the Roman Baths: Isolation of oleaginous species and analysis of the FAME profile for biodiesel production. Table S1. Primer pairs used for amplification of either U16S (cyanobacteria) or U18S (eukaryotic algae) gene regions. Second primer sets created if sequences were too short (i.e. <1000bp). * modified from (Taton et al. 2003), **(Cuvelier et al. 2008). Table S2. Composition of bath thermal waters (Great Bath (GB) and Kings Bath (KB)) compared to historical measurements from the kings spring (Kellaway 1991). Analysis performed by Severn Trent Services, unless stated all data in mg/L. Table S3. Confocal microscope images of C.emersonii and roman bath isolates stained with nile red after cultivation at 20°C temperatures with and without nitrogen starvation. Table S4. Confocal microscope images of C.emersonii and roman bath isolates stained with nile red after cultivation at 30°C temperatures with and without nitrogen starvation. Table S5. Confocal microscope images of C.emersonii and roman bath isolates stained with nile red after cultivation at 40°C temperatures with and without nitrogen starvation. Figure S1. Microcoleus chthonoplastes full 16S rDNA sequence. Figure S2. Oscillatoria sancta full 16S rDNA sequence. Table S6. FAME% profiles of the microbes cultured at 20°C. Table S7. FAME profiles (%) of the microbes cultured at 30°C. Table S8. FAME profiles (%) of the microbes grown at 40°C, rows given in italics are partial FAME profiles based on the limited amount of material that was available. Figure S3. Dry weight of the isolates grown under nitrogen enriched conditions at 20°C. Figure S4. Dry weight of the isolates grown under nitrogen enriched conditions at 30°C. Figure S5. Dry weight of the isolates grown under nitrogen enriched conditions at 40°C. [file 2191-0855-3-9-S1.docx]

Supplementary material: Bioprospecting the thermal waters of the Roman Baths: Isolation of oleaginous species and analysis of the FAME profile for biodiesel production

Holly D. Smith-Bädorf,^a^ Christopher J. Chuck,^b^ Kirsty R. Mokebo,^c^ Heather MacDonald^d^ Matthew G. Davidson^c^ and Rod J. Scott,^a^*

^a^ Department of Biology and Biochemistry, University of Bath, Bath, BA2 7AY, United Kingdom

^b^ Department of Chemical Engineering, University of Bath, Bath, BA2 7AY, United Kingdom

^c^ Department of Chemistry, University of Bath, Bath, BA2 7AY, United Kingdom

^d^ Department of Applied Sciences, University of the West of England, Bristol, BS16 1QY, United Kingdom

Tel: +44 (0)01225 383437

Fax: +44 (0)1225 386779

Email: R.J.Scott@bath.ac.uk

**Table S1**: Primer pairs used for amplification of either U16S (cyanobacteria) or U18S (eukaryotic algae) gene regions. Second primer sets created if sequences were too short (i.e. <1000bp). * (modified from Taton *et al.*, 2003), **(Cuvelier *et al.*, 2008)

| **Isolate** | **1^st^ primer pair (name/Tm)** | **2^nd^ primer pair (name/Tm)** |
| --- | --- | --- |
| *Os* | AGAGTTTGATCCTGGCTCAG (U16SF/54.0°C)*  ACGGCTACCTTGTTACGACTT (U16SR/56.2°C)* | U16SF  TATCTAATCCCATTCGCTCC (GBU16SR#2/54.6°C) |
| *Mc* | AGAGTTTGATCCTGGCTCAG (U16SF/54.0°C)*  ACGGCTACCTTGTTACGACTT (U16SR/56.2°C)* | U16SF  AACCACATACTCCACCGC (KBU16SR#2/53.1°C) |
| *Ml* | AGAGTTTGATCCTGGCTCAG (U16SF/54.0°C)*  ACGGCTACCTTGTTACGACTT (U16SR/56.2°C)* | - |
| *K* sp. | ACCTGGTTGATCCTGCCAG (U18SF/57.7°C)**  TGATCCTTCTGCAGGTTCAC (U18SR/55.4°C)** | - |
| *Cs* | ACCTGGTTGATCCTGCCAG (U18SF/57.7°C)**  TGATCCTTCTGCAGGTTCAC (U18SR/55.4°C)** | ATTACCCAATCCTGATACGG (RBSU18SF~2/54.2°C)  U18SR |
| *Ct* | AGAGTTTGATCCTGGCTCAG (U16SF/54.0°C)*  ACGGCTACCTTGTTACGACTT (U16SR/56.2°C)* | - |
| *H* sp. | ACCTGGTTGATCCTGCCAG (U18SF/57.7°C)**  TGATCCTTCTGCAGGTTCAC (U18SR/55.4°C)** | ATCATTCAAGTTTCTGCCC (RBDU18SF#2/52.1°C)  U18SR |

**Table S2** Composition of bath thermal waters (Great Bath (GB) and Kings Bath (KB)) compared to historical measurements from the kings spring (Kellaway, 1991). Analysis performed by Severn Trent Services, unless stated all data in mg/L.

| **Elements** | **1874** | **1888** | **1936** | **1961** | **1979** | **1986** | **2011 GB** | **2011 KB** |
| --- | --- | --- | --- | --- | --- | --- | --- | --- |
| B |  |  |  |  | 0.59 |  | 0.47 | 0.51 |
| Ca | 377 | 402 | 392 | 392 | 382 | 390 | 394 | 421 |
| Cu |  |  |  |  | 0.002 |  | 0.002 | 0.003 |
| Fe |  |  |  |  | 0.88 |  | 0.25 | 0.39 |
| Mg | 47 | 52 | 51 | 54 | 53 | 58 | 51 | 53 |
| Mn |  |  |  |  | 0.068 |  | 0.135 | 0.140 |
| Mo |  |  |  |  | <0.1 |  | <0.002 | <0.002 |
| K | 39 | 31 | 15.4 | 15.7 | 17.4 | 18.1 | 20.1 | 20.8 |
| Na | 129 | 135 | 177 | 174 | 183 | 228 | 196 | 203 |
| Cl | 280 | 277 | 274 | 276 | 287 | 335 | 332 | 330 |
| NO_3_^-^N |  |  |  |  | <0.1 |  |  |  |
| HCO_3_ | 86 | 88 | 193 | 216 | 192 | 187 |  |  |
| SiO_2_ |  |  |  |  | 20.6 (Si) |  | 42.99 | 42.27 |
| SO_4_ | 869 | 1061 | 1001 | 1021 | 1032 | 1030 | 978 | 977 |
| **BOD** |  |  |  |  |  |  | <1 | <1 |
| **pH** |  |  |  |  | 6.65 |  |  |  |
| **Temp °C** | 46 | 47 | 49 | 48 | 45.3 | 43.5 | 39.0 | 45.0 |

**Table S3** Confocal microscope images of *C.emersonii* and roman bath isolates stained with nile red after cultivation at 20°C temperatures with and without nitrogen starvation.

| **Species** | **N sufficient** | **N starvation** |
| --- | --- | --- |
| *Ce* | 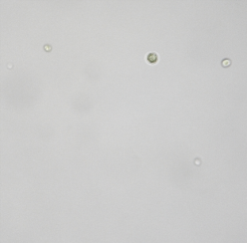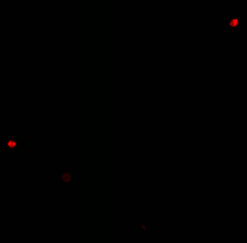 | 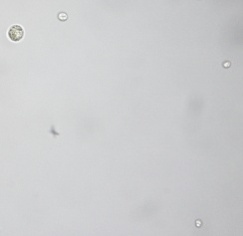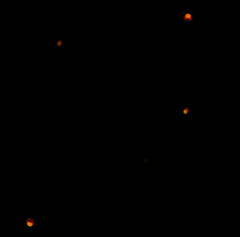 |
| *Cs* | 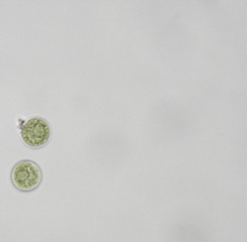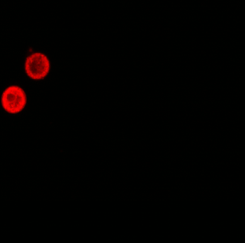 | 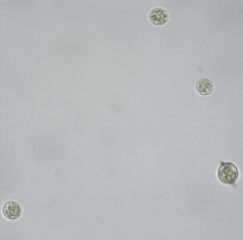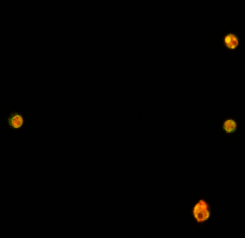 |
| *K* sp. | 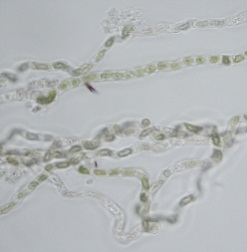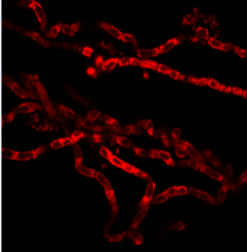 | 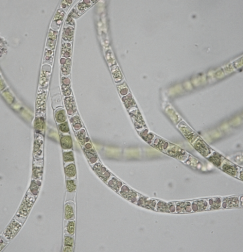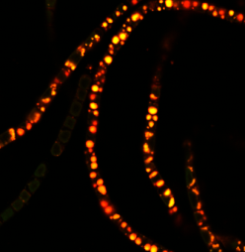 |
| *H* sp. | 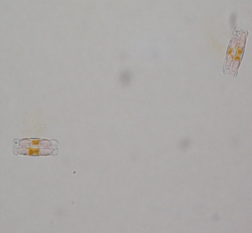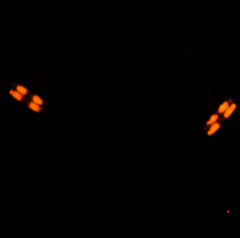 | 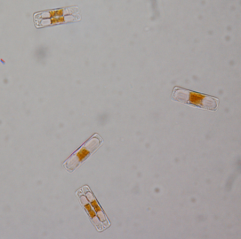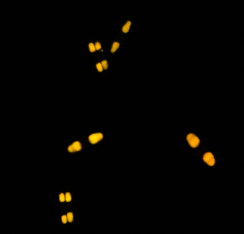 |
| *Ct* | 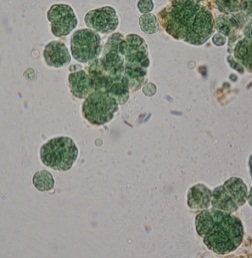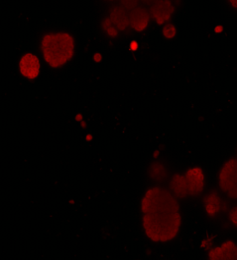 | 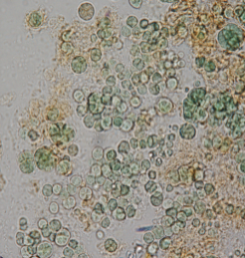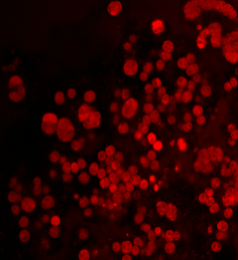 |
| *Mc* | 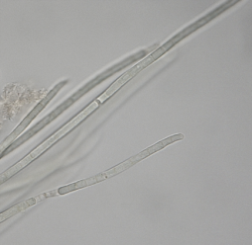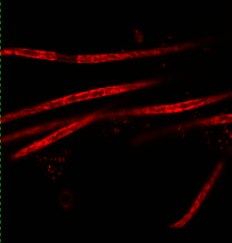 | 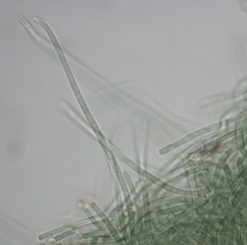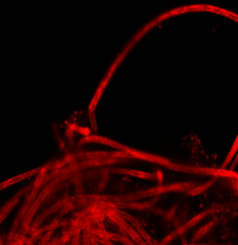 |
| *Ml* | 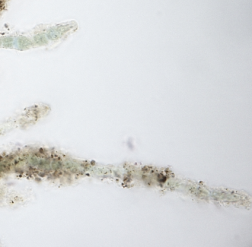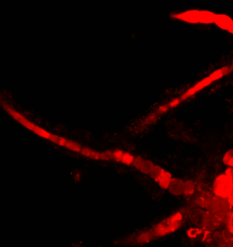 | 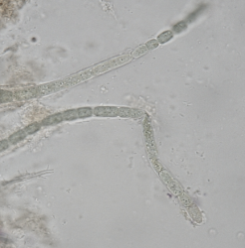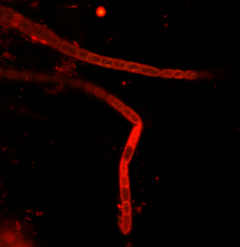 |
| *Os* | 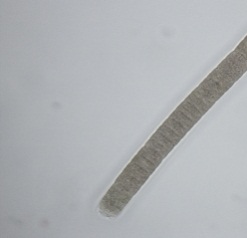 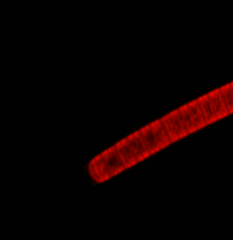 | 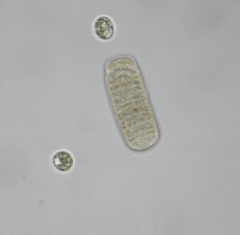 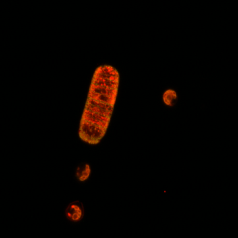 |

**Table S4** Confocal microscope images of *C.emersonii* and roman bath isolates stained with nile red after cultivation at 30°C temperatures with and without nitrogen starvation.

| **Species** | **N sufficient** | **N starvation** |
| --- | --- | --- |
| *Ce* | 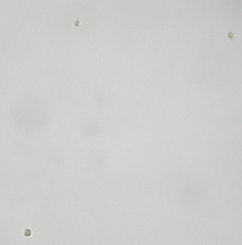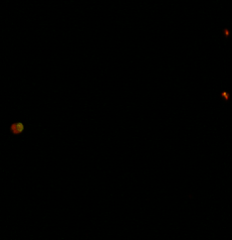 | 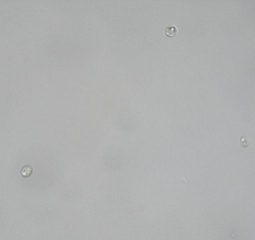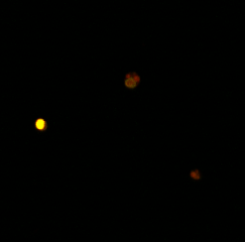 |
| *Cs* | 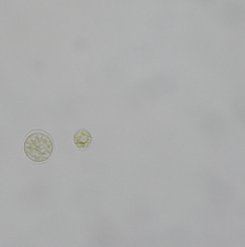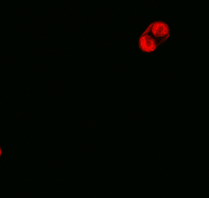 | 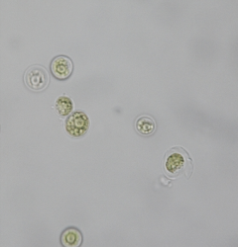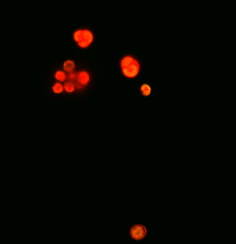 |
| *K* sp. | 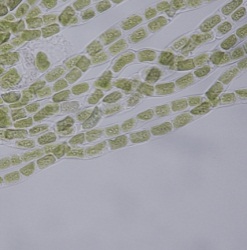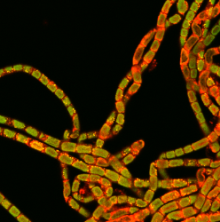 | 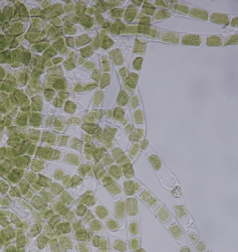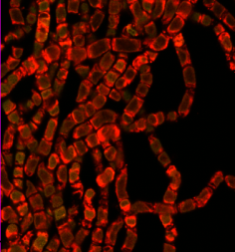 |
| *H* sp. | 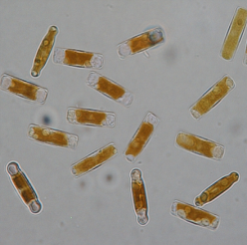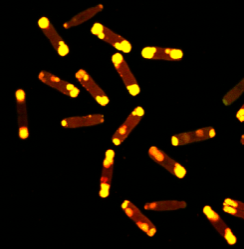 | 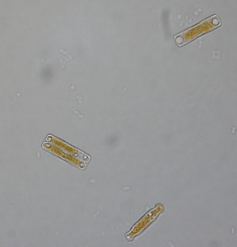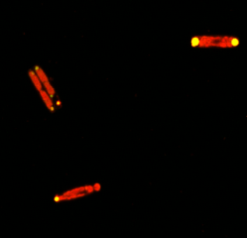 |
| *Ct* | 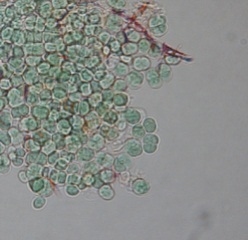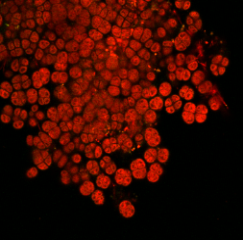 | 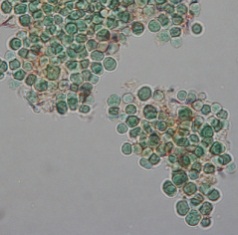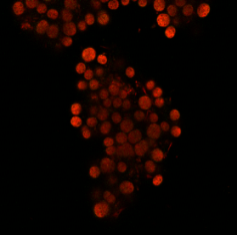 |
| *Mc* | 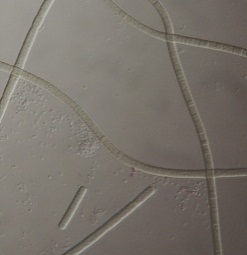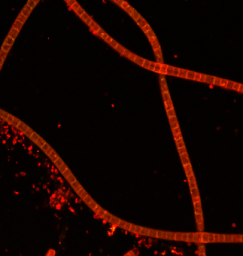 | 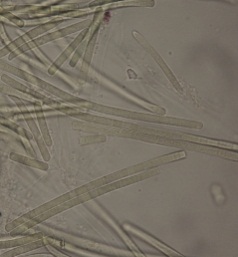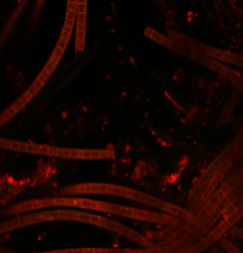 |
| *Ml* | 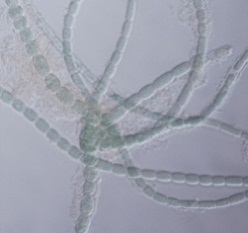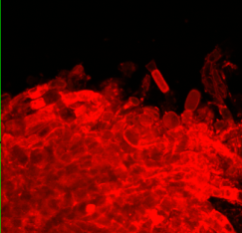 | 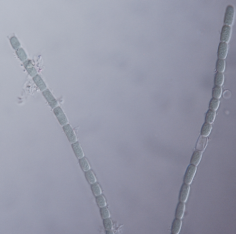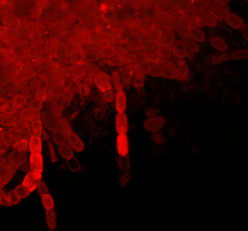 |
| *Os* | 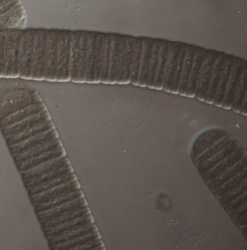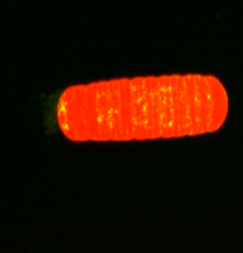 | 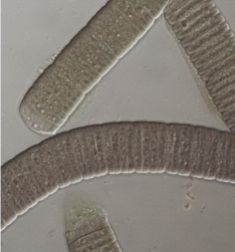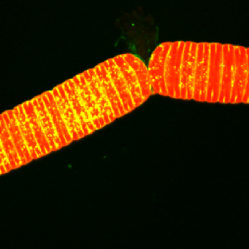 |

**Table S5** Confocal microscope images of *C.emersonii* and roman bath isolates stained with nile red after cultivation at 40°C temperatures with and without nitrogen starvation.

| **Species** | **N sufficient** | **N starvation** |
| --- | --- | --- |
| *Ce* | No growth | No growth |
| *Cs* | No growth | No growth |
| *K* sp. | No growth | No growth |
| *H* sp. | No growth | No growth |
| *Ct* | 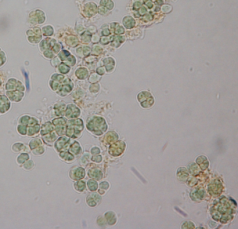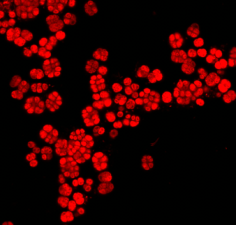 | 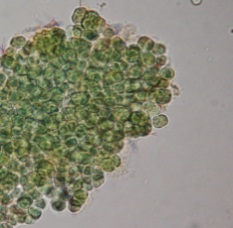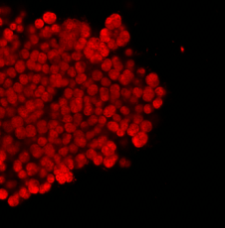 |
| *Mc* | 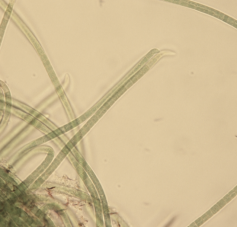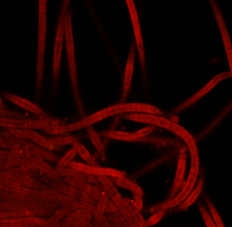 | 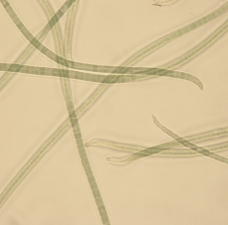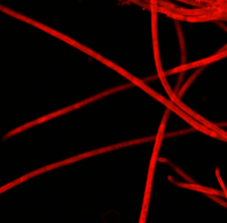 |
| *Ml* | 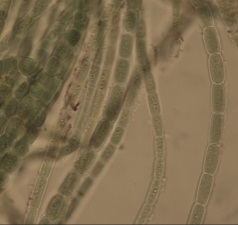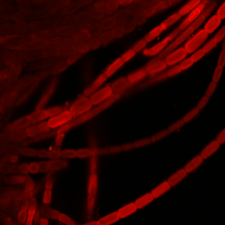 | 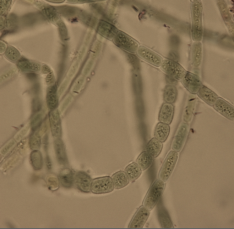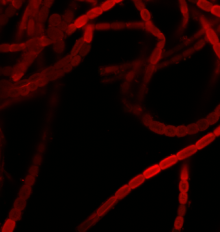 |
| *Os* | Poor growth | Poor growth |

NTTTGATCCTGGCTCAGGATGAACGCTGGCGGTCTGCTTAACACATGCAAGTCGAACGGGCTCTTCGGAGCTAGTGGCGGACGGGTGAGTAACGCGTGAGAATCTGCCCACAGGAGGGGGACAACCACTGGAAACGGTGGCTAATACCCCATAAGCCGANGAGGAATCCGCCTGTGGAGCTCGCGTCTGATTAGCTAGTAGGTGAGGTAAGAGCTTACCTAGGCGATCAGTANNGCTGGTCTGAGAGNNNNNGATNGAGCAGNCCACACTGGNGACTGAGACACGGCCCAGACTCCTACGGGAGGCAGCAGTGGGGAATTTTCNCGNNCAATGGGCGAAAGCCTGACGGAGCCAGANCCGCGTGAGGGGAAGAAGGCCNTTTGGGTTGTAAACCTCTTTTCTCAGGAAGAAGAAAGTGACGTACCTGAGGAATCAGCCTCNGGCTNACTNCCGTGCCCAGCAGCCGCGGTAAGACGGAGGAGGCAAGCGTTATCCGGAATTATTGGGCGTAAAGCGTCCGTAGGTGGTGATTCAAGTCTGCTGTCAAAACCCAGGGCTTAACTCTGGATCGGCAGTGGAAACTGAATGACTAGAGTGCGGCAGGGGCAGAGGGAATTCCCGGTGTAGCGGTGAAATGCGTAGAGATCGGGAAGAACACCGGTGGCGAAAGCGCTCTGCTGGGCCGCGACTGACACTCACAGGACGAAAGCTAGGGGAGCGAATGGGATTAGATACCCCAGTAGTCCTAGCTGTAAACGATGGAAACTAGGTGTGGCCTGTATCGACCCGGGCCGTGCCGGAGCTAACGCGTTAAGTTTCCCGCCTGGGGAGTACGCACGCAAGTGTGAAACTCAAAGGAATTGACGGGGGCCCGCACAAGCGGTGGAGTATGTGGTTTAATTCGATGCAACGCGAAGAACCTTACCAGGGCTTGACATGTCGCGAATCCCGGTGAAAGCTGGGAGTGCCTTCGGGAGCGCGAACACAGGTGGTGCATGGCTGTCGTCAGCTCGTGTCGTGAGATGTTGGGTTAAGTCCCGCAACGAGCGCAACCCTCGTCCTTAGTTGCCAGCATTCAGATGGGAACTCTAGGGAGACTGCCGGTGACAAACCGGAGGAAGGTGGGGATGACGTCAAGTCAGCATGCCCCTTACGTCCTGGGCGACACACGTACTACAATGGTTGGGACAAAGGGTAGCCAAGCCGCGAGGACGAGCCAATCCCATAAACCCAGCCACAGTTCAGATCGCAGGCTGCAACTCGCCTGCGTGAAGGAGGAATCGCTAGTAATTGCCGGTCAGCATACGGCAGTGAATCCGTTCCCGGGCCTTGTACACACCGCCCGTCACACCATGGGAGCTGGCCACGCCCGAAGTCGTTACTCTAACCNTNGCGGAGGNGNNGCCGNAGN

**Fig. S1** *Microcoleus chthonoplastes* full 16S rDNA sequence

NGNTNNNNTNNNTACGGGTTAGAGTAATGACTTCGGGCGTGGCCAACTTCCATGGTGTGACGGGCGGTGTGTACAAGGCCCGGGAACGGATTCACCGCAGTATGCTGACCTGCGATTACTAGCGATTCCGACTTCATGCAGGCGAGTTGCAGCCTGCAATCTGAACTGAGGCCTGGTTTTAGGGATTGGCTCACCCTCGCGGGTTGGCTACCCTCTGTCCAGACCATTGTAGTACGTGTGTAGCCCAGGGCGTAAGGGGCATGCTGACTTGACGTCATCCCCACCTTCCTCCGGTTTGTCACCGGCAGTCTCTTCAGAGTGCCCAACTTAATGCTGGCAACTAAAGACGAGGGTTGCGCTCGTTGCGGGACTTAACCCAACATCTCACGACACGAGCTGACGACAGCCATGCACCACCTGTGTTCGCGCTCCCGAAGGCACTCCAAGGTTTCCCCTAGATTCGCGACATGTCAAGCCCTGGTAAGGTTCTTCGCGTTGCATCGAATTAAACCACATACTCCACCGCTTGTGCGGGCCCCCGTCAATTCCTTTGAGTTTCACACTTGCGTGCGTACTCCCCAGGCGGGATACTTAACGCGTTAGCTACGGCACTGCCCGGGTCGATACAGGCAACACCTAGTATCCATCGTTTACAGCTAGGACTACTGGGGTATCTAATCCCNNNNGCTCCCCTAGCTTNCGTCCCTGAGTGTCAGTTCTGGTCTAGCCAAGNNCTTTCNNCNCCGATGTTCTTCCCGATCTCTACGCATTTCACCGCTACACCGGGAATTCCCTTGGCCCCTACCAGACTCTAGCTTTGNAGTTTCCACTGCCTAACCAGAGTTAAGCCCTNGGGNNTTGACAGCNNNNNANNNNNNTCTGGTCTAGCCAGCGCTTTCGCCACCGATGTTCTTCCCGATCTCTACGCATTTCACCGCTACACCGGGAATTCCCTTGGCCCCTACCAGACTCTAGCTTTGTAGTTTCCACTGCCTAACCAGAGTTAAGCCCTGGGTTTTGACAGCAGACTTACATGGCCACCTGCGGACGCTTTACGCCCAATCATTCCGGATAACGCTTGCCTCCTCCGTATTACCGCGGCTGCTGGCACGGAGTTAGCCGAGGCTGATTCCTTTGGTACCGTCAGTACTTCTTCCCAAAGAAAAGGGGTTTACAACCCTAGAGCCTTCCTCCCCCACGCGGTCTTGCTCCGTCAGGCTTTCGCCCATTGCGGAAAATTCCCCACTGCTGCCTCCCGTAGGAGTCTGGGCCGTGTCTCAGTCCCAGTGTGGCTGCTCATCCTCTCAGACCAGCTACTGATCGTCGCCTTGGTAAGCTTTTACCTTACCAACTAGCTAATCAGACGCGAGCTCATCTCCAGGCAATTAATCTTTCACCCGTAGGCTTATCCGGGATTAGCAGAAGTTTCCCTCTGTTGCCCAGACCAGAAGGCAGATTCTCACGCGTTACTCACCCGTCCGCCACTAACTCCGAAGAGTCCGTTCGACTTGCATGTGTTAAGCAGACCGCCAGCGTTCATNNNGAGCCAGGATCAAACN

**Fig. S2** *Oscillatoria sancta* full 16S rDNA sequence

**Table S6** FAME % profiles of the microbes cultured at 20 °C

|  | ***Ce*** | | ***K* sp.** | | ***Cs*** | | ***H* sp.** | | ***Os*** | | ***Mc*** | | ***Ml*** | | ***Ct*** | |
| --- | --- | --- | --- | --- | --- | --- | --- | --- | --- | --- | --- | --- | --- | --- | --- | --- |
|  | +N | -N | +N | -N | +N | -N | *+N* | *-N* | *+N* | *-N* | +N | -N | +N | -N | +N | -N |
| **C14:0** | 0.2 | 0.6 |  | 1.1 | 0.3 | 0.8 | *5.1* | *5.6* | *5.2* | *4.1* | 2.8 | 2.7 | 4.0 | 3.4 | *1.6* | *5.2* |
| **C15:0** | 0.1 | 0.1 |  |  |  | 0.1 |  | *0.9* |  |  |  |  |  |  |  |  |
| **C16:0** | 18.9 | 18.0 | 62.5 | 25.5 | 15.7 | 18.4 | *48.1* | *44.3* | *45.1* | *58.4* | 59.8 | 68.7 | 54.4 | 55.1 | *20.3* | *51.8* |
| **C16:1** | 1.4 | 1.4 | 7.7 | 0.7 | 5.7 | 4.4 | *35.2* | *32.4* |  |  |  |  | 11.1 | 8.8 | *15.1* | *21.1* |
| **C18:0** | 1.7 | 4.3 | 20.0 | 5.7 | 2.3 | 5.2 | *9.3* | *10.9* | *49.7* | *37.5* | *37.4* | *28.5* | *30.5* | *32.7* |  | *18.9* |
| **C18:1** | 28.5 | 38.5 | 3.8 | 2.7 | 28.1 | 30.0 |  | *4.7* |  |  |  |  |  |  |  |  |
| **C18:2** | 10.2 | 6.9 | 5.9 | 46.2 | 8.9 | 6.2 |  |  |  |  |  |  |  |  |  |  |
| **C18:3** | 34.7 | 23.0 |  | 15.4 | 32.8 | 24.8 |  |  |  |  |  |  |  |  |  |  |
| **C20:1** | 0.0 | 0.0 |  |  | 0.4 |  |  |  |  |  |  |  |  |  |  |  |
| **C20:4** | 0.0 | 0.0 |  |  |  |  |  |  |  |  |  |  |  |  |  |  |
| **C16:2** | 3.0 | 2.0 |  | 0.6 | 3.1 | 2.3 | *2.3* | *1.26* |  |  |  |  |  |  | *62.9* | *3.0* |
| **C16:3** | 0.7 | 4.8 |  | 2.1 | 0.1 | 5.7 |  |  |  |  |  |  |  |  |  |  |
| **C16:4** | 0.4 | 0.5 |  |  | 2.2 | 1.0 |  |  |  |  |  |  |  |  |  |  |
| **C18:4** | 0.2 | 0.0 |  |  | 0.2 | 1.0 |  |  |  |  |  |  |  |  |  |  |

**Table S7** FAME profiles (%) of the microbes cultured at 30 °C

|  | ***Ce*** | | ***K* sp.** | | ***Cs*** | | ***H* sp.** | | ***Os*** | | ***Mc*** | | ***Ml*** | | ***Ct*** | |
| --- | --- | --- | --- | --- | --- | --- | --- | --- | --- | --- | --- | --- | --- | --- | --- | --- |
|  | +N | -N | +N | -N | +N | -N | +N | -N | +N | -N | +N | -N | +N | -N | +N | -N |
| **C14:0** | 0.3 | 0.5 | 2.4 |  | 0.4 | 0.5 | 5.0 | 4.3 | 30.2 | 20.5 |  | 0.8 | 1.8 |  | 5.0 | 4.7 |
| **C15:0** | 0.0 | 0.6 |  |  | 0.3 | 0.2 |  |  |  |  |  |  |  |  | 0.8 |  |
| **C16:0** | 17.0 | 13.1 | 60.3 | 54.8 | 17.2 | 19.3 | 50.0 | 49.6 | 33.3 | 25.6 | 66.7 | 49.2 | 42.9 | 42.5 | 42.7 | 55.8 |
| **C16:1** | 2.1 | 3.8 |  | 3.7 | 4.7 | 3.3 | 20.3 | 18.5 | 12.6 | 2.3 |  |  |  | 23.3 | 24.3 | 26.6 |
| **C18:0** | 3.1 | 2.5 | 33.0 | 24.2 | 2.1 | 5.0 | 21.5 | 23.6 | 12.6 | 9.1 | 33.3 | 11.2 | 23.5 | 23.7 | 11.5 | 12.9 |
| **C18:1** | 8.7 | 21.1 |  |  | 19.4 | 16.5 |  |  |  |  |  | 8.7 | 31.7 | 10.5 |  |  |
| **C18:2** | 9.1 | 12.8 | 4.3 | 13.6 | 11.8 | 10.8 | 3.2 |  | 10.9 | 5.6 |  | 5.0 |  |  |  |  |
| **C18:3** | 46.6 | 29.2 |  |  | 28.7 | 32.6 |  |  | 0.4 | 34.6 |  | 16.0 |  |  | 14.4 |  |
| **C20:1** | 0.0 | 1.0 |  |  | 0.5 |  |  |  |  |  |  |  |  |  |  |  |
| **C20:4** | 1.4 | 0.0 |  |  |  |  |  |  |  |  |  |  |  |  |  |  |
| **C16:2** | 1.7 | 3.1 |  | 3.8 | 4.6 | 3.6 |  | 4.0 |  |  |  |  |  |  | 1.3 |  |
| **C16:3** | 0.9 | 3.5 |  |  | 7.8 | 8.3 |  |  |  |  |  |  |  |  |  |  |
| **C16:4** | 6.7 | 5.9 |  |  | 0.9 |  |  |  |  |  |  |  |  |  |  |  |
| **C18:4** | 2.4 | 2.8 |  |  | 1.6 |  |  |  |  |  |  | 9.0 |  |  |  |  |

**Table S8** FAME profiles (%) of the microbes grown at 40 °C, rows given in italics are partial FAME profiles based on the limited amount of material that was available.

|  | ***Os*** | | ***Mc*** | | ***Ml*** | | ***Ct*** | |
| --- | --- | --- | --- | --- | --- | --- | --- | --- |
|  | *+N* | *-N* | +N | -N | +N | -N | *+N* | *-N* |
| **C14:0** |  |  |  |  |  |  |  |  |
| **C15:0** |  |  |  |  |  |  |  |  |
| **C16:0** | *54.4* | *56.1* | 49.0 | 49.1 | 38.5 | 42.2 | *56.7* | *54.8* |
| **C16:1** |  |  |  |  | 31.4 | 23.7 |  |  |
| **C18:0** | *45.6* | *43.9* | 28.7 | 12.4 | 15.5 | 17.0 | *43.3* | *43.3* |
| **C18:1** |  |  | 6.2 | 9.6 | 14.5 | 16.1 |  |  |
| **C18:2** |  |  |  | 8.9 |  |  |  |  |
| **C18:3** |  |  | 16.0 | 20.0 |  |  |  |  |
| **C20:1** |  |  |  |  |  |  |  |  |
| **C20:4** |  |  |  |  |  |  |  |  |
| **C16:2** |  |  |  |  |  |  |  |  |
| **C16:3** |  |  |  |  |  |  |  |  |
| **C16:4** |  |  |  |  |  |  |  |  |
| **C18:4** |  |  |  |  |  |  |  |  |

**Fig. S3** Dry weight of the isolates grown under nitrogen enriched conditions at 20 °C

**Fig. S4** Dry weight of the isolates grown under nitrogen enriched conditions at 30 °C

**Fig. S5** Dry weight of the isolates grown under nitrogen enriched conditions at 40 °C
