# Supplementary material for: Bioprospecting the thermal waters of the Roman baths: isolation of oleaginous species and analysis of the FAME profile for biodiesel production
Source: AMB Express. 2013 Jan 31;3:9. doi: 10.1186/2191-0855-3-9 (PMC3571919; doi:10.1186/2191-0855-3-9)
Supplement: Additional file 2: Table 2 — Temperature tolerance experiments comparing growth of C.emersonii and Roman Bath isolates. Growth was assessed visually by comparing samples across the temperature ranges. – no growth, + poor growth, ++ good growth, +++ vigorous growth, NT not tested. [file 2191-0855-3-9-S2.doc]

**Table 2** Temperature tolerance experiments comparing growth of *C.emersonii* and Roman Bath isolates. Growth was assessed visually by comparing samples across the temperature ranges.

| **Species** | **20°C** | **25°C** | **30°C** | **35°C** | **40°C** | **45°C** | **50°C** | **55°C** | **60°C** |
| --- | --- | --- | --- | --- | --- | --- | --- | --- | --- |
| *C.emersonii* | ++ | +++ | ++ | + | - | - | - | - | - |
| *C.saipanensis* | ++ | +++ | ++ | + | - | - | - | - | - |
| *Klebsormidium* sp*.* | ++ | +++ | ++ | + | - | - | - | - | - |
| *Hantzschia* sp*.* | ++ | NT | ++ | NT | - | NT | NT | NT | NT |
| *C.thermalis* | ++ | NT | ++ | NT | ++ | NT | NT | NT | NT |
| *M.chthonoplastes* | + | + | ++ | +++ | +++ | ++ | - | - | - |
| *M.laminosus* | + | + | ++ | +++ | +++ | ++ | - | - | - |
| *O.sancta* | ++ | ++ | ++ | ++ | + | + | - | - | - |

– no growth, + poor growth, ++ good growth, +++ vigorous growth, NT not tested.
